# Spreading-Factor Usage and Airtime in Urban LoRaWAN Traffic: Passive Gateway Monitoring with Machine Learning

**DOI:** 10.3390/s26216855

**Published:** 2026-10-29

**Authors:** Nuri Furkan Koçak, Ahmet Mehmet Karadeniz, László T. Kóczy

**Affiliations:** 1 Department of Electronics and Automation, Kırıkkale Vocational School, Kırıkkale University, Kırıkkale 71450, Türkiye; nfkocak@kku.edu.tr; 2 Doctoral School of Multidisciplinary Engineering Sciences, Széchenyi István University, 9026 Győr, Hungary; 3 Department of Information Technology, Széchenyi István University, 9026 Győr, Hungary; koczy@tmit.bme.hu

**Keywords:** LoRaWAN, passive monitoring, spreading factor, airtime, join requests, machine learning

## Abstract

Evidence on whether spreading-factor (SF) usage follows link quality in passively observed urban Long-Range Wide-Area Network (LoRaWAN) traffic remains limited. This study analyzes 54,893 Long-Range (LoRa) frames decoded over 30.2 days by a receive-only gateway in Ankara, Türkiye, using only physical-layer metadata and unencrypted message types. Join requests formed 73.44% of frames and 77.73% of airtime. A gradient-boosting classifier separated SF7 from SF12 frames with area under the precision–recall curve (PR-AUC) = 0.9376; on a subsequent 24.9-day external test period, the F1 score was 0.7161, alongside a persistent frequency-offset shift. This discrimination relied largely on SF7 frames without a valid LoRaWAN uplink header; within LoRaWAN uplinks, link-quality variables outperformed frame size combined with frequency offset (PR-AUC 0.5010 versus 0.1129), also externally. At a 10 dB margin, 20.81% of SF12 join requests but only 0.31% of SF12 data uplinks had a signal-to-noise ratio sufficient for a lower SF; join requests accounted for 11.08 of 11.14 percentage points of counterfactual airtime reduction. Unsupervised clustering yielded six signal-quality regimes aligned with message class. Data-uplink SF usage is consistent with link-quality-driven assignment, whereas join requests dominate airtime; separating message classes was necessary for passive interpretation.

## 1. Introduction

Long-Range Wide-Area Network (LoRaWAN) networks operated by different parties share the same unlicensed sub-GHz channels. On these channels, transmissions with longer airtime occupy the channel for longer under duty-cycle limits and reduce the share of frames that can be delivered as the number of transmitters grows [1,2], so airtime is the resource that every network within range draws on. An operator planning a deployment, or auditing an existing one, typically has no access to the network servers or application payloads of the neighboring networks that load the same channels. Gateway-side passive observation addresses this situation: a receive-only gateway records physical-layer (PHY) and medium access control (MAC) metadata for every frame it decodes, regardless of network affiliation [3,4].

The spreading factor (SF) governs this airtime. Long-Range (LoRa) modulation encodes each symbol as a chirp spanning $2^{\mathrm{SF}}$ chips, transmitted at a chip rate equal to the channel bandwidth [5], which is 125 kHz for LoRa uplinks at data rates DR0–DR5 (SF12–SF7) in the European 863–870 MHz band (EU868) [6]. Each unit increase in SF therefore doubles the symbol duration, $T_{\mathrm{sym}}=2^{\mathrm{SF}}/BW$, where BW is the channel bandwidth, while the occupied bandwidth remains unchanged [5]. The longer symbol provides processing gain: the signal-to-noise ratio (SNR) required for demodulation falls by 2.5 dB per SF step, from −7.5 dB at SF7 to −20 dB at SF12 [7], at the cost of a twofold increase in symbol duration and an approximately twofold increase in time-on-air (ToA, referred to as airtime below) per step [1,7].

Adaptive data rate (ADR) mechanisms are intended to assign the lowest SF that the measured link margin allows [8,9]. Such an assignment cannot, however, be assumed for every observed frame. ADR is optional, and its network-server-side algorithm is not explicitly defined in the LoRaWAN specification. In the 2020 implementation of The Things Network, devices at the slowest data rate (DR0) in applications without downlink data requirements were found never to receive a data-rate change, regardless of link quality. Moreover, any data rate may be used in the join procedure, and this data rate becomes the initial data rate of the device [9]. In the gateway-only setting described above, whether SF usage follows link quality must therefore be assessed from the received metadata. If SF assignment follows link quality as intended, SF should be predictable from the SNR received at the serving gateway(s), and frames transmitted at high SF should arrive with SNR close to the corresponding demodulation limit.

Prior work has examined LoRaWAN link behavior from three directions, summarized in Table 1. Measurements with the authors’ own or otherwise known transmitters have quantified long-term reliability, outdoor coverage, indoor link quality and dense indoor network performance [10,11,12,13]; these studies establish propagation and reliability characteristics but, by design, do not observe an ambient device population. Passive monitoring studies have observed that population in several cities [3,4,14] and reported traffic, channel, security and duty-cycle statistics, relying on MAC-header fields such as the device address (DevAddr), but they did not report the SF of individual frames against their link margin. Machine learning studies, surveyed in [15], have addressed SF allocation, largely in simulation [16], propagation modeling for link-budget analysis with known transmitters [17], and anomaly or intrusion detection based on payload sensor values [18] or on per-device models keyed to the device address [19], and k-means clustering has been used to profile devices from the radio and network behavior of packets stored in an operator database [20].

Nevertheless, empirical evidence on whether SF usage in ambient urban traffic tracks link quality remains limited, particularly with respect to evidence that can be obtained from gateway metadata alone [3,4,14]. That SF7 frames arrive at higher SNR than SF12 frames is expected from the modulation itself; what remains unknown is how far actual SF usage departs from this expectation and what that departure costs. These observations highlight three unresolved issues: whether the SF of an ambient frame is better explained by its link quality or by its message type and device- or application-level attributes; how much airtime is consumed by frames whose link margin would permit a lower SF; and whether a model trained on a short observation window remains valid as the device population changes. Addressing these issues requires a framework combining multi-week real-world observation, stratification by message class, a counterfactual airtime analysis, dependence-aware statistics, ablation-based attribution of classifier performance, and evaluation on an unseen external test period.

Gateway-only observation defines the data source and acts as a constraint: the analysis uses only what a receive-only gateway reports. Data minimization is a design principle, not a novel privacy-preserving technique: payload bytes and device identifiers are excluded at the parsing stage, which limits but does not eliminate re-identification risk (Section 6.3). Message-type stratification, a counterfactual airtime analysis, and supervised and unsupervised learning are the analytical methods applied to the resulting metadata. The analysis therefore concerns what can be inferred from gateway metadata once payload bytes and device identifiers have been removed.

To address these issues, the present study analyzes 30.2 days of ambient traffic captured by a receive-only gateway in Ankara, Türkiye, and assesses temporal generalization on a subsequent, unseen external test period of 24.9 days. The principal contributions of this study are as follows:

(i) A 30.2-day real-world dataset of 54,893 decoded LoRa frames with their message class, analyzed with day-block bootstrap inference that accounts for temporal dependence between frames and publicly released together with the analysis script [21].
(ii) An ablation-based attribution, extended by message class, showing that the apparent contribution of frame size and frequency offset to SF7/SF12 discrimination arises largely from LoRa frames without a valid LoRaWAN uplink header, whereas, within LoRaWAN uplinks, link-quality variables outperform frame size combined with frequency offset, both in the analysis window and on the external test period.
(iii) A frame-level over-provisioning analysis by message class showing that SF12 data uplinks are rarely over-provisioned at a 10 dB margin, whereas join requests account for most observed airtime and nearly all counterfactual airtime savings.
(iv) An evaluation of temporal generalization on an unseen 24.9-day external test period, documenting the F1 score (harmonic mean of precision and recall) on that period alongside a persistent frequency-offset shift and a lower SF7 prevalence.
(v) An SF-free clustering and anomaly-detection analysis whose signal-quality regimes are linked to message class and over-provisioning, providing a descriptive segmentation of the observed traffic.

## 2. Experimental Setup

**Figure 1:**
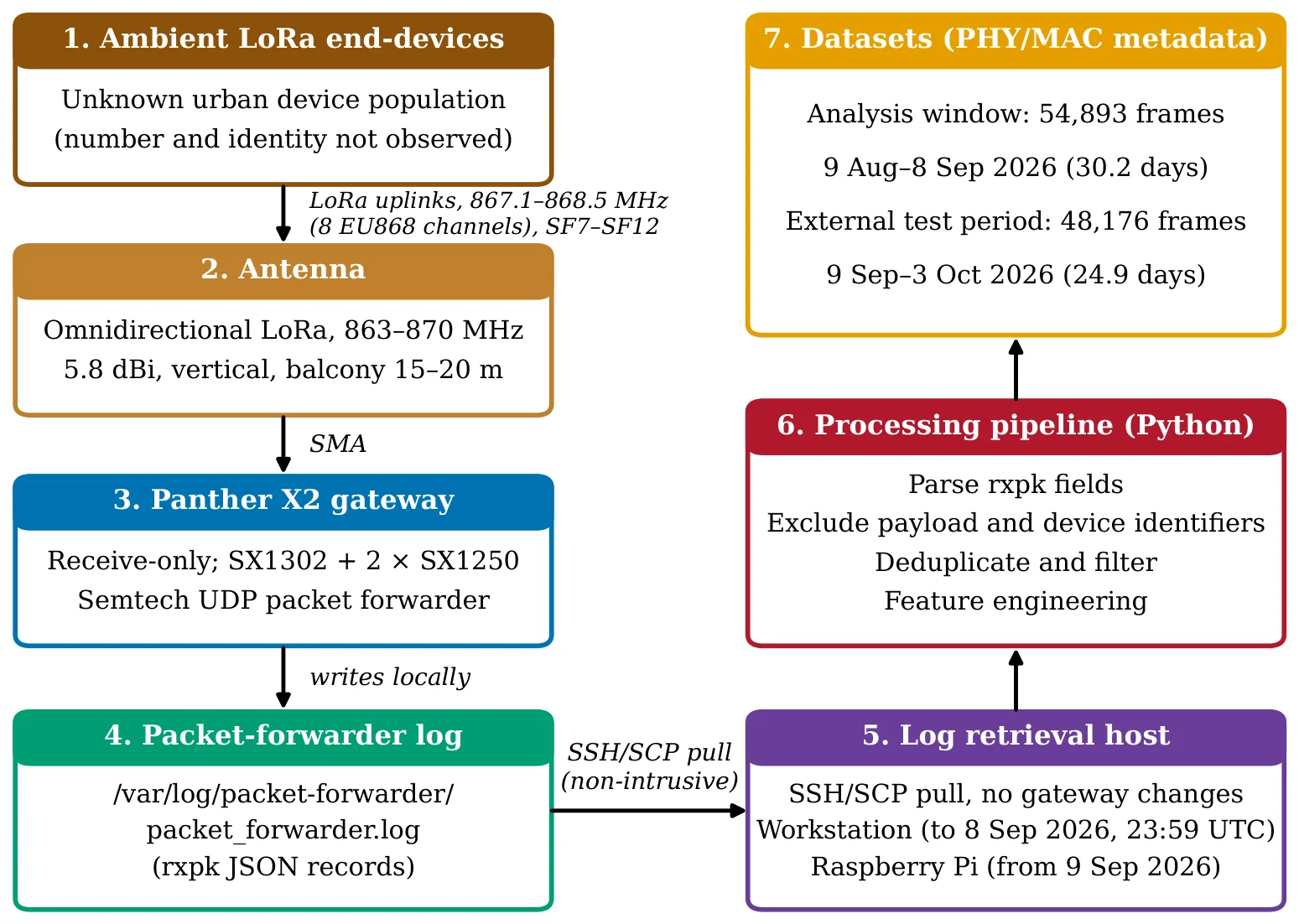
Passive Long-Range (LoRa) observation and data acquisition architecture. Abbreviations: European 863–870 MHz band (EU868); JavaScript Object Notation (JSON); Secure Copy Protocol (SCP); SubMiniature version A (SMA); Secure Shell (SSH); User Datagram Protocol (UDP); Coordinated Universal Time (UTC).

The overall measurement and data acquisition architecture is illustrated in Figure 1. The setup follows a passive observation approach in which ambient LoRa transmissions are received by the gateway, locally logged by the packet forwarder, and subsequently retrieved and processed on a separate host, where payload bytes and device identifiers are removed at parsing. The main hardware components, deployment conditions, and data acquisition procedure are described in the following subsections.

### 2.1. LoRaWAN Gateway Hardware

The measurement platform was a commercially available LoRaWAN gateway (Panther X2; E-Sun Electronics, Hong Kong, China). Its radio section comprises a Semtech SX1302 (Semtech Corporation, Camarillo, CA, USA) multi-channel concentrator paired with two SX1250 front-ends supporting the EU868 channel plan; the deployed global_conf.json confirms eight channels distributed across the two front-ends, five on radio 0 and three on radio 1. The host processor is a Rockchip RK3566 (Rockchip Electronics, Fuzhou, China) with 3.81 GB random-access memory (RAM) (confirmed via /proc/cpuinfo and /proc/meminfo), running a Debian GNU/Linux-based operating system (kernel 4.19.193, aarch64). The gateway’s factory-configured received signal strength indicator (RSSI) offset (rssi_offset =−215.4) was applied by the concentrator firmware to all reported RSSI values; no additional manual calibration was performed. The concentrator receives on the standard eight-channel EU868 configuration (867.1–868.5 MHz) and exposes frame-level metadata through the Semtech User Datagram Protocol (UDP) packet forwarder, logged locally to /var/log/packet-forwarder/packet_forwarder.log.

Although the platform is primarily marketed as a Helium-network mining appliance, the Helium software, including its blockchain-layer Proof-of-Coverage subsystem, was disabled throughout the study, so the gateway transmitted no Proof-of-Coverage beacons. The packet-forwarder log operates independently of network-layer join or blockchain state. It records all frames successfully decoded on the concentrator’s configured channels, regardless of network affiliation; frames outside the configured channel plan or below the receiver’s demodulation threshold are not captured.

### 2.2. Antenna and Urban Deployment

An omnidirectional LoRa antenna (Omni-5.8dBi; Boardoza, Istanbul, Türkiye; 863–870 MHz, manufacturer-rated 5.8 dBi gain, 650 mm length) was connected directly to the gateway’s SubMiniature version A (SMA) port via a short (<0.5 m) coaxial pigtail, replacing the stock antenna, and mounted in a vertical orientation on an open balcony of a residential building in Ankara, Türkiye, at approximately 15–20 m above street level. No independent gain calibration or receiver RSSI-offset calibration was performed beyond the manufacturer’s factory specification; reported RSSI and SNR values therefore reflect the combined response of the antenna, cable, and concentrator front-end as configured, rather than a metrologically calibrated absolute measurement. The deployment site was selected to reflect a realistic, non-optimized urban installation rather than a purpose-built high-sensitivity monitoring station.

### 2.3. Data Acquisition Architecture

Gateway access was established over the local area network via Secure Shell (SSH; password-authenticated, non-default administrative account). Until 8 September 2026, 23:59 Coordinated Universal Time (UTC), log files were retrieved from a workstation at irregular intervals (typically one to three times per day) using a lightweight PowerShell (Microsoft Corporation, Redmond, WA, USA) script (fetch_lora_log.ps1) that performs a Secure Copy Protocol (SCP) transfer of the remote log file to a timestamped local archive, without modifying or deleting data on the gateway. Thereafter, retrieval was migrated to a single-board computer (Raspberry Pi 3 Model B; Raspberry Pi Ltd., Cambridge, UK) running the same procedure on a fixed schedule. This pull-based design introduced no traffic on the observed band and no configuration changes on the gateway. Because the gateway log grows cumulatively between retrievals, retrieval cadence affects neither the frames captured nor their metadata; the only log rotation of the campaign (26 September 2026, external test period) was recovered from the rotated file. All timestamps are host-clock UTC; local time in Ankara is UTC+3 throughout the study, without daylight-saving changes.

During the initial deployment phase, a transient fault was identified in the vendor-supplied watchdog service (pantherx-watchdog.service), which entered a restart loop upon failing to resolve an update endpoint over IPv6 while the local network path supported only IPv4. The fault was resolved by forcing IPv4 resolution in the watchdog’s download routine; no changes were made to the packet-forwarder configuration, channel plan, or antenna. No frames were logged between the study start (14:48 UTC) and 18:26:55 UTC on 9 August, an interval most likely corresponding to the physical installation of the antenna; the analysis window therefore begins at the first valid frame. Within the analysis window (Section 3.2), the packet-forwarder process restarted once (30 August 2026, 10:52 UTC) and frame capture was interrupted for approximately 18 min on 6 September 2026; the longest inter-frame gap in the window was 41 min. The external test period contains two unplanned gateway reboots (12 and 21 September 2026) and one 7.7 h outage (27 September 2026); these are retained as part of the operational conditions under which the models were evaluated.

### 2.4. Regulatory and Privacy Considerations

The gateway was operated as a receive-only instrument. No network server was attached to the packet forwarder, and no downlink, acknowledgment or beacon transmissions were scheduled during the study, so the deployment emitted no radio-frequency energy in the observed band and the antenna gain affected reception only. Observation was limited to frames that nearby end-devices broadcast in a license-exempt band; payloads were not decrypted, and the raw packet-forwarder logs, which contain the encoded PHY payload, were held only on the gateway and the retrieval hosts, used solely for parsing and not released; the processed dataset retains neither payloads nor device identifiers. Consistent with the data-minimization principle (Article 5(1)(c) of Regulation (EU) 2016/679) [22], only the metadata fields required for the analysis were extracted (Section 3.1).

The measurement site is in Türkiye. The processed dataset contains no payload content, device identifiers or other data that directly identify a natural person within the meaning of the Personal Data Protection Law No. 6698 [23]; the retained data consist only of radio-signal and frame-level technical parameters, which may nonetheless permit linkage of transmissions from persistent devices (Section 6.3). Observation was passive and no radio-frequency signal was transmitted; the observed band lies within the 863–870 MHz range designated for short-range devices under the regulation on radio equipment exempt from frequency assignment issued under Electronic Communications Law No. 5809 [24]. The study involved no transmission, no interaction with end-devices or their operators and no human participants; the institutional review statement is therefore reported as not applicable.

## 3. Dataset Construction and Preprocessing

### 3.1. Frame-Level Data

Each retrieved log entry corresponds to a Semtech PUSH_DATA record containing a JavaScript Object Notation (JSON)-encoded rxpk object with one entry per received frame. From each entry, the following fields were extracted: reception timestamp (host clock, UTC), internal concentrator timestamp (tmst), logical channel index (chan), radio front-end index (rfch), center frequency (freq, MHz), modulation type (modu), data rate identifier encoding SF and bandwidth (datr), coding rate (codr), signal-only and total RSSI (rssis, rssi, dBm), SNR (lsnr, dB), carrier frequency offset (foff, Hz; hereafter frequency offset), PHY payload length (size, bytes), and cyclic redundancy check (CRC) status (stat).

The raw data field, containing the base64-encoded PHY payload, was not retained in the processed dataset. This field contains the MAC header and, for data frames, the encrypted frame payload together with the device address (DevAddr), which is transmitted unencrypted and can identify a device without decryption [3]; join requests are not encrypted and carry the device’s join extended unique identifier (JoinEUI) and device extended unique identifier (DevEUI) in clear. Exclusion was enforced at the parsing stage, so that payload bytes and device identifiers are absent from the processed dataset and were not used in any analysis reported here.

During parsing, only the 3-bit message type (MType) of the unencrypted MAC header, which identifies the frame category but not the device, was decoded from the first byte of the PHY payload and retained; the remaining payload bytes, including the DevAddr, were discarded. Each frame was assigned to one of three message classes: join requests (join-request MType and the mandatory 23-byte length), data uplinks (unconfirmed or confirmed data-up MType), and frames without a valid LoRaWAN uplink header (all other cases). The last class consisted almost entirely of 2479 SF7 frames received at 868.3 MHz, all but one with 15- or 17-byte payloads. Their header bits would denote downlink message types, which a gateway configured for uplink reception would not normally decode, and include frames labeled as join accepts with an invalid length; these frames are therefore most likely transmissions of LoRa applications that do not use the LoRaWAN uplink format. The three-bit header code was available for all frames of the analysis window and of the external test period; it was interpreted as an MType only for frames consistent with the LoRaWAN uplink format.

### 3.2. Analysis Window and External Test Period

The analysis window spans 9 August 2026, 18:26:55 UTC (the first valid frame after deployment) to 8 September 2026, 23:59:59 UTC; with the last frame received at 23:59:24 UTC, it covers 725.5 h (30.23 days). Its end was fixed by the acquisition procedure rather than by any analysis result: it is the last instant before log retrieval moved from the workstation to the single-board computer, so the entire window was collected under one unchanged acquisition setup. Frames received between 9 September 2026, 00:00 UTC, and 3 October 2026, 20:57 UTC, form an external test period of 24.9 days (48,176 frames), that is, a later period at the same site with the same equipment rather than an independent site. This period was not used for model selection or hyperparameter choice. It served for the primary temporal evaluation reported in Section 5.7, which was followed by additional post hoc message-class sensitivity and distribution-shift analyses on the same period.

### 3.3. Data Cleaning

Records predating the study start (9 August 2026, 14:48 UTC, the confirmed onset of the Ankara deployment) were excluded to prevent contamination from residual log entries associated with the gateway’s prior configuration state. Deduplication was performed using a composite key (timestamp, concentrator timestamp, channel, frequency, RSSI, SNR, frame size) to eliminate redundant records arising from overlapping log retrievals, since the source log grows cumulatively between acquisitions. All records exhibited a valid CRC. One frame (24 August 2026, 19:20:55 UTC) reporting SF6, coding rate OFF and a zero-byte payload was excluded because it does not correspond to a valid LoRaWAN uplink. A further 2038 frames in the analysis window (3.71%, all SF12) reported signal-only RSSI values of −141 dBm or lower, which lie at the reporting floor of that field. These frames passed CRC and were retained; repeating the primary classification without them changed the test F1 from 0.7917 to 0.7918.

### 3.4. Feature Engineering

The categorical datr field (e.g., SF12BW125) was decomposed into an ordinal SF variable and a bandwidth variable; all frames used a bandwidth of 125 kHz and coding rate 4/5. Local hour of day and local calendar date (UTC+3) were derived for the diurnal and weekly analyses. The base feature set for supervised learning comprises seven gateway-reported variables: RSSI, SNR, signal-only RSSI (rssis), frequency offset, frame size, center frequency and channel index. Signal-only RSSI is closely related to the other two signal variables: for frames with negative SNR, rssis differed from the sum of RSSI and SNR by a mean of 0.75 dB (standard deviation (SD) 0.76 dB), and by at most 1.5 dB for 89.8% of frames.

To examine temporal signal behavior, eleven temporal features (nine window-based and two cyclical) were computed causally, using only frames received strictly before the current frame: the number of frames received on all channels in the preceding 5, 15 and 60 min; the number received on the same channel in the preceding 15 min; the mean and standard deviation of SNR over the preceding 15 and 60 min; the mean RSSI over the preceding 60 min; and the sine and cosine of the local hour of day. Window statistics that were undefined, such as a standard deviation over fewer than two frames or windows with too few preceding frames, mainly after gaps, were filled with medians computed on the training period only. The message class was not used as a classifier input; it served to define subsets for sensitivity analyses and to stratify the over-provisioning analysis.

The processed records of the analysis window (54,893) and of the external test period (48,176) are released as version 2 of the dataset described in the Data Availability Statement.

## 4. Analysis Methods

### 4.1. Dependence-Aware Statistical Inference

Consecutive frames may originate from the same transmitters and therefore cannot be assumed independent, so frame-level tests can overstate precision. Confidence intervals (CIs) were therefore obtained by a block bootstrap [25] using non-overlapping local calendar days as blocks, resampled with replacement (percentile intervals; 2000 resamples for the tests in this subsection, 1000 for the main classification metrics and for the frame-level comparison described below, and 500 for the ablation and external-test tables), which preserves within-day dependence. The chronological test set and the external test period comprise 12 and 25 local days, respectively, so these intervals are approximate indicators of day-to-day variability; they resample the predictions of fixed models and do not capture training variability. The SF7–SF12 SNR difference is reported with both the naive Welch interval and the day-block interval. To quantify the effect of this dependence on the classification results, the LightGBM test metrics and 13 paired F1 and PR-AUC contrasts from Section 5.4 and Section 5.5 (between the three ensembles, the three key ablation contrasts in the test and external periods, and the link-quality contrast within LoRaWAN uplinks) were also computed with a frame-level bootstrap that resamples individual frames (1000 resamples), and the frame-level and day-block intervals were compared.

Diurnal asymmetry was tested on the per-day share of frames received between 06:00 and 17:59 local time, using a one-sample Wilcoxon signed-rank test against 50% over the 30 complete days in the window. Weekday and weekend activity were compared on daily frame counts (Mann–Whitney *U* test; bootstrap confidence interval (CI) of the difference, resampling days within each group), treating days rather than frames or hours as the unit of analysis. Pearson correlations and Cohen’s *d* are reported descriptively, without adjustment for dependence.

### 4.2. Supervised Spreading-Factor Classification

The classification task is not intended to recover SF, which the gateway already reports in the datr field. It serves as a diagnostic of how SF is assigned. If SF were set from the link quality observed at this gateway, a model given only link-quality variables would reproduce it, and adding device- or application-related variables would add little. The gap between these two cases therefore indicates how far SF usage departs from assignment driven by the locally observed link quality; it cannot distinguish static configuration from adaptation to a different serving gateway (Section 6.1). The simplest mathematical model of link-quality-driven assignment is a threshold rule that labels a frame SF7 when its SNR exceeds the SF7 demodulation limit of −7.5 dB [7]; this rule is reported alongside logistic regression (LR) baselines on SNR, RSSI, and both.

The primary task distinguishes SF7 from SF12 frames, which together account for 89.5% of frames and represent the two ends of the SF range, where the expected SNR separation (12.5 dB between demodulation limits) is largest. Multi-class formulations are reported as complementary analyses (Section 5.6). Frames were ordered by timestamp, and the first 70% of SF7/SF12 frames (34,387 frames, 4067 SF7; 9 August 18:26 to 29 August 10:54 UTC) formed the training set, while the remaining 30% (14,738 frames, 835 SF7; 29 August 10:54 to 8 September 23:59 UTC) formed the chronological test set, so that no test frame precedes any training frame.

Six classifiers were compared: logistic regression, a support vector machine (SVM) with radial basis function (RBF) kernel, Random Forest [26], Extra Trees [27], extreme gradient boosting (XGBoost) [28] and light gradient boosting machine (LightGBM) [29]. Hyperparameters were selected by three-fold time-series cross-validation restricted to the training period, in which each validation fold follows its training fold in time; candidate settings were scored by the area under the precision–recall curve (PR-AUC), which is appropriate for the minority SF7 class and was computed as average precision (scikit-learn average_precision_score). For logistic regression and the SVM, standardization was part of the model pipeline and was therefore fitted within each training fold. Tree ensembles were tuned by 12 random draws from the search spaces listed in Section 5.4; logistic regression and the SVM were tuned by grid search, the latter on every fourth training frame to limit run time, so its comparison with the other classifiers is approximate. The final model was selected by cross-validation score alone; the test set played no part in model or hyperparameter selection. Data-driven selection was preferred over fixed default settings because the choice among otherwise interchangeable operators can change computational cost and outcome quality substantially, as shown for aggregation operators in fuzzy-signature-based robot path planning [30].

All models used class-balanced weighting (XGBoost: positive-class weight equal to the training class ratio). Performance on the test set was summarized by balanced accuracy, precision, recall, F1 and Matthews correlation coefficient (MCC) at the default decision threshold, and by the area under the receiver operating characteristic curve (ROC-AUC) and PR-AUC; the PR-AUC of a no-skill classifier equals the SF7 prevalence (0.0567 in the chronological test set). 95% CIs for F1, ROC-AUC and PR-AUC were obtained by day-block bootstrap of the test set (1000 resamples). The sensitivity of the primary result to the 2038 frames at the signal-only RSSI floor was assessed by refitting without them (Section 3.3).

To attribute the discrimination to individual features, the selected model was refitted with its tuned hyperparameters on ten feature subsets (Section 5.5), including subsets without frequency offset, without frame size, without both, and with link-quality variables only; logistic regression was refitted on the same subsets. Hyperparameters tuned on the full feature set were reused; because the subsets were not retuned, the magnitude of the ablation differences is conditional on the hyperparameters selected for the full model. Differences in PR-AUC for three key contrasts (all features versus without frequency offset, all features versus without frame size, and frequency offset combined with frame size versus link-quality variables) were assessed by paired day-block bootstrap (1000 resamples). Because the frequency offset is measured against the gateway’s own reference oscillator and differed between the two radio front-ends, the selected model was also refitted on radio-1 frames only and with the frequency offset centered on the training median of each front-end. Permutation importance on the test set (decrease in PR-AUC over ten permutations) was computed as a complementary indicator; it is less affected than impurity-based importance by a preference for continuous or high-cardinality predictors [31], but it can misattribute importance among correlated predictors such as SNR and signal-only RSSI [32], so the ablation is used as the primary basis for attribution.

The classification and the three key contrasts were repeated on LoRaWAN uplinks only (join requests and data uplinks) and on join requests only, using the same chronological 70/30 rule within each subset, so that the test portions of the subsets need not begin at the same time; for the external test period, the model was refitted on the same subset of the entire analysis window and evaluated on the corresponding subset of the external period.

A six-class model (SF7–SF12) and a three-class model (SF7, SF8–11, SF12) were trained on all frames in the window with an analogous chronological 70/30 split of all frames (cut at 29 August 2026, 13:34 UTC), using Random Forest and LightGBM with fixed class-balanced configurations, with and without the frequency offset, and evaluated by macro-averaged F1; the three-class model was additionally evaluated on the external test period. The contribution of the temporal features (Section 3.4) was assessed by refitting the selected model on the base features, on the base features plus temporal features, on temporal features alone, and on temporal features with the base features except the frequency offset.

Temporal generalization was assessed in two ways. First, weekly forward-chaining evaluation trained each model on weeks 1,…,k of the window and tested it on week k+1 (k=1,…,4), using fixed configurations (library defaults with class-balanced weighting, except Random Forest: 200 trees, maximum depth 10, minimum split size 5; Extra Trees: 200 trees); the SVM was omitted from this analysis because of its run time. Second, the selected model and logistic regression were refitted on the entire analysis window and evaluated once on the external test period, overall and per week.

Distribution shift of SNR, RSSI, frequency offset and frame size in each external week relative to the analysis window was quantified by the two-sample Kolmogorov–Smirnov (KS) statistic, both over all frames, which also reflects changes in SF composition, and within SF12 frames only, as a descriptive indicator of feature-distribution shift (virtual concept drift) [33]; the statistic ranges from 0 for identical distributions to 1 for non-overlapping ones. To examine the frequency-offset shift, the daily median frequency offset of SF12 join requests and of SF12 data uplinks (days with at least 50 frames, analysis window and external period combined) was related to the daily mean outdoor air temperature at the gateway location, taken from the hourly model output of the Open-Meteo service [34], and to the daily mean processor temperature logged by the gateway at 2 min intervals from 2 September 2026. Spearman correlations were computed after removal of a linear time trend from both series.

### 4.3. Unsupervised Analysis

Clustering used six PHY-layer features that do not contain the SF label (RSSI, SNR, signal-only RSSI, frequency offset, frame size and frequency), standardized to zero mean and unit variance. Because SNR and signal-only RSSI are strongly correlated (r=0.982; Section 5.2), link quality carries extra weight in the Euclidean distance; unlike the k-means profiling of packets stored in an operator database [20], no device identity is used, and the clusters are interpreted descriptively. SF was excluded from the clustering input and from the selection of the number of clusters. *k*-means was evaluated for k=2 to 10 using the silhouette score [35] (computed on a fixed random sample of 5000 frames), the Davies–Bouldin index [36] and the Calinski–Harabasz index [37]. The number of clusters was not fixed in advance; the selection rule was fixed before the search range was extended to k=10: the value of *k* favored by at least two of the three indices, or, failing agreement, the value favored by the silhouette score. The upper bound of k=10 was set so that the optimum, if any, could fall in the interior of the search range.

Stability was assessed by the adjusted Rand index (ARI) [38] between partitions from five random initializations, and between the global partition and partitions refitted separately on each week. The resulting clusters were cross-tabulated post hoc against SF and against the over-provisioning indicator of Section 4.4, so that each signal-quality regime could be given an operational interpretation. To test whether the clustering adds information to the SF7/SF12 classification, the selected model was refitted with cluster membership, or with the distances of each frame to the six cluster centroids, added to its features. For the chronological test set, the standardization and *k*-means were fitted on the training period only; for the external test period, they were fitted on the full analysis window. Differences in PR-AUC were assessed by paired day-block bootstrap (1000 resamples).

Isolation Forest [39] was applied to the same standardized features with contamination values of 0.5%, 1%, 2% and 5%; profiles are reported for 2%. The analysis was repeated with the temporal features added, and the overlap of flagged sets was measured by the Jaccard index.

### 4.4. Spreading-Factor Over-Provisioning and Airtime

The airtime of each frame, $T_{\mathrm{frame}}$, was computed from the standard LoRa formula [7,40]:

(1) $$T_{\mathrm{frame}}=\left(n_{\mathrm{pre}}+4.25+n_{\mathrm{pay}}\right)T_{\mathrm{sym}},$$

(2) $$n_{\mathrm{pay}}=8+\max\;\left(\left\lceil \frac{8PL-4\;\mathrm{SF}+28+16\;\mathrm{CRC}-20H}{4\;(\mathrm{SF}-2DE)}\right\rceil \left(CR+4\right),\;0\right),$$

where $n_{\mathrm{pay}}$ is the number of payload symbols, $T_{\mathrm{sym}}=2^{\mathrm{SF}}/BW$, BW=125 kHz, $n_{\mathrm{pre}}=8$ preamble symbols, PL is the PHY payload length in bytes, CR=1 (coding rate 4/5), H=0 (explicit header), CRC=1, and DE=1 for SF11 and SF12 (low-data-rate optimization) and 0 otherwise. A frame was classified as over-provisioned at link margin *M* if a lower SF *s* satisfied $\mathrm{SNR}\geq L_{s}+M$, where $L_{s}$ is the demodulation SNR limit of SF*s* (−7.5, −10, −12.5, −15, −17.5 and −20 dB for SF7–SF12 [7]). These nominal limits are those of the SX1276 transceiver and serve as standardized reference thresholds for comparison across SFs rather than as calibrated sensitivity limits of the deployed gateway; the SX1302 concentrator decoded a portion of frames below them (28.44% of SF7 and 30.02% of SF12 frames in the analysis window), so the limits, and the resulting estimate, are conservative in this respect. Because a uniform shift of all limits is equivalent to a change in *M*, the four margins evaluated below also indicate how sensitive the estimate is to uncertainty in the limits.

The counterfactual airtime assigns each frame the lowest SF meeting this condition, never raising any frame’s SF, and is reported for M=0, 5, 10 and 15 dB, for all frames and separately for join requests and data uplinks. The Semtech-recommended algorithm uses a margin parameter usually set to 5 dB [9]; 10 dB is used here as the primary, more conservative value because a single-frame SNR lacks the multi-frame maximum on which that algorithm operates. Because the network-server ADR algorithm recommended by Semtech and adopted by common network servers evaluates the maximum SNR over the last 20 received uplinks rather than a single frame [9], this frame-level counterfactual is an indicator of potential airtime savings, not a prediction of what an ADR mechanism would assign.

### 4.5. Software

All analyses were performed in Python 3.14.7 (Python Software Foundation, Wilmington, DE, USA) with scikit-learn 1.9.0 [41], XGBoost 3.4.1, LightGBM 4.7.0, pandas 3.0.5 [42], NumPy 2.5.3 [43] and SciPy 1.18.1 [44], with all random seeds fixed at 42; the complete statistical and machine learning analysis of the released frame-level files is implemented as a single script published with the dataset.

## 5. Results

### 5.1. Dataset Statistics

**Figure 2:**
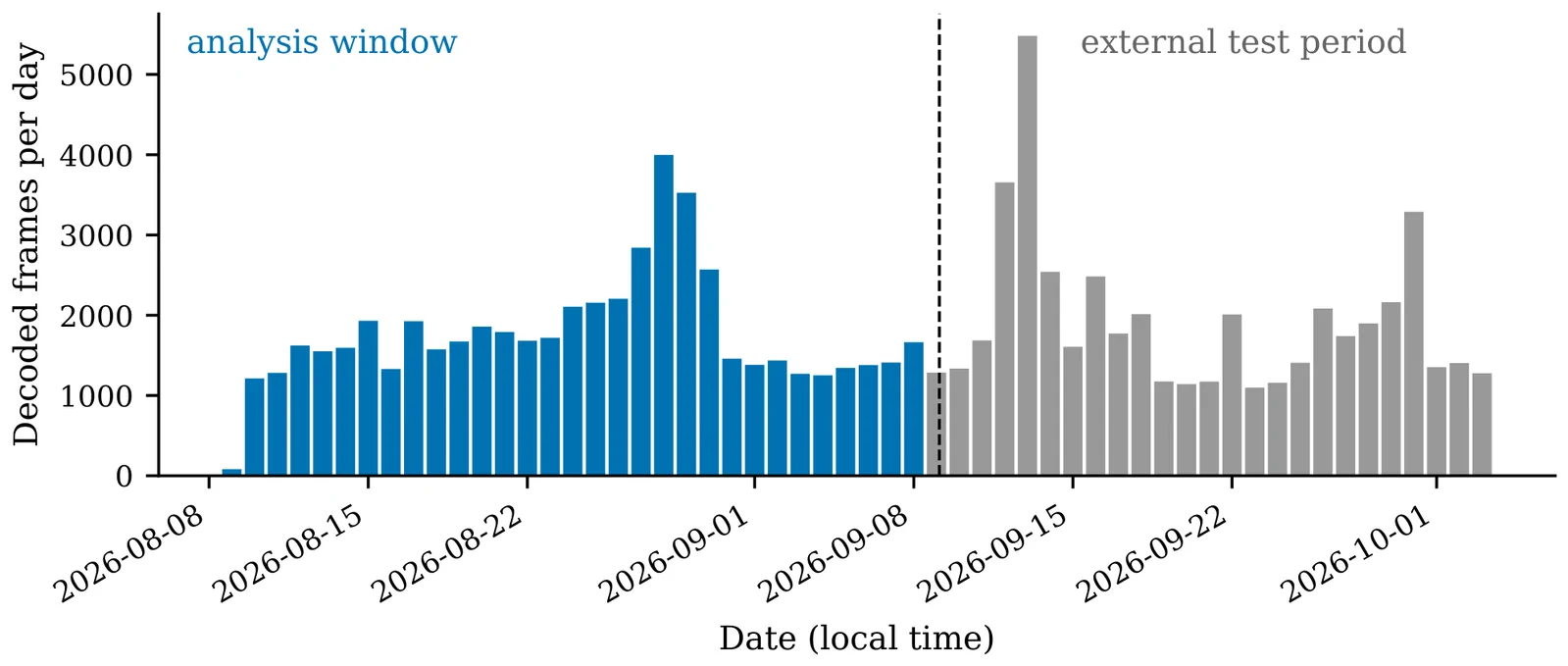
Daily decoded-frame counts over the measurement campaign (local dates). Blue: analysis window; gray: external test period.

The analysis window contains 54,893 decoded LoRa frames over 725.5 h, a mean of 75.66 frames per hour. On the 30 complete local days, daily counts ranged from 1213 to 3998 frames (mean 1825.1). Figure 2 shows the daily counts for the entire campaign, including the external test period; the bar for 9 September (local) is shown in gray because only 59 of its frames belong to the analysis window. Exact daily counts for the analysis window, broken down by SF group, are given in Table A1 (Appendix A); those for the external test period are included in the released dataset. SF composition changed within the window: frames at SF8–SF11 rose from 17–92 per day between 10 and 24 August to 671–1502 per day between 27 and 30 August, and SF7 frames fell from 95–492 per day in August to 18–79 per day in September (Table A1). These counts reflect frames successfully decoded by the gateway, not total ambient transmission volume, since frames outside the configured channels, below receiver sensitivity, or lost to collisions are not observed.

### 5.2. Signal Quality and Traffic

**Figure 3:**
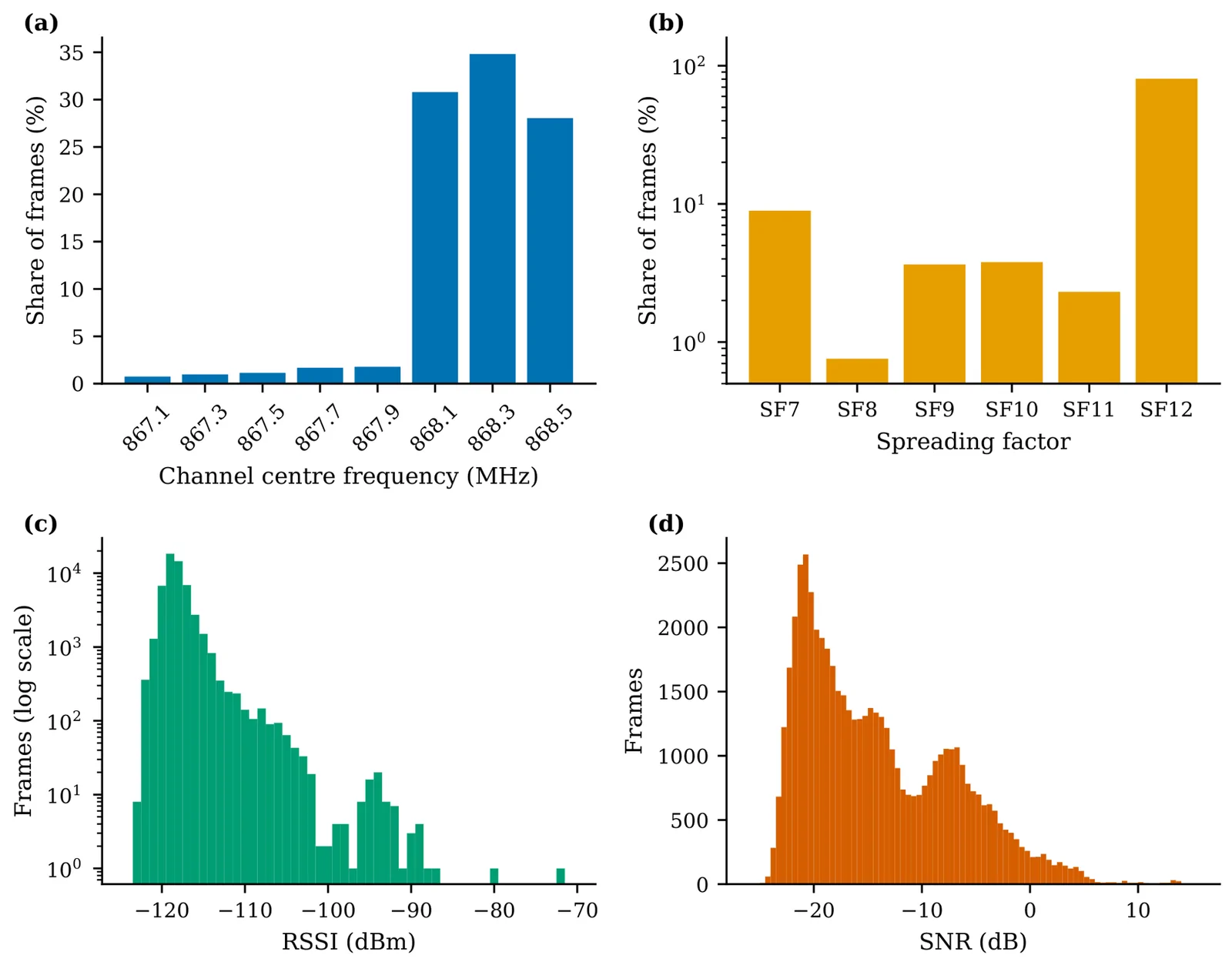

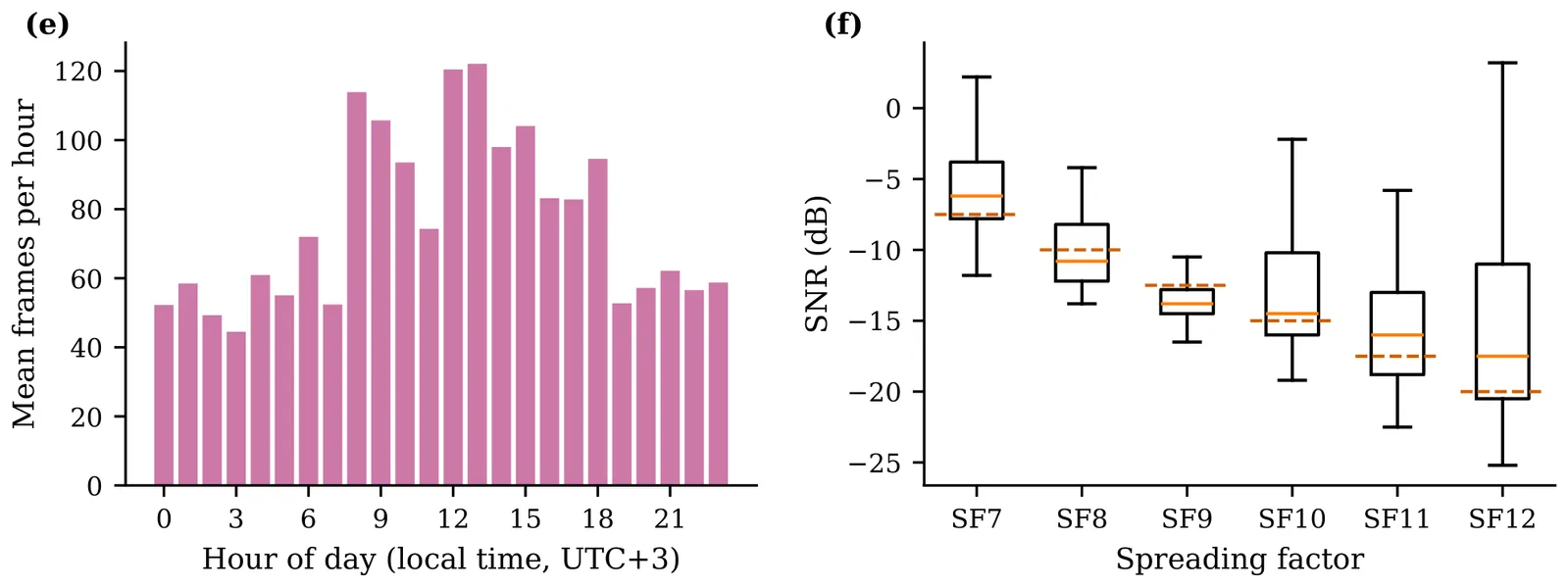
Observed traffic in the analysis window (*n* = 54,893): (**a**) channel; (**b**) SF; (**c**) RSSI; (**d**) SNR; (**e**) mean frames per hour by local hour; (**f**) SNR by SF, with demodulation limits (dashed). Bar colors only distinguish the panels. In (**f**), boxes show the interquartile range, whiskers extend to 1.5 times the interquartile range, and solid orange lines mark the medians.

RSSI was concentrated in a narrow band (median −118 dBm, interquartile range (IQR) [−119,−118] dBm); only 80 frames exceeded −100 dBm, with a maximum of −72 dBm (Figure 3c). SNR was multimodal, with density peaks near −20.9, −15.0 and −7.6 dB and minor maxima above +8 dB (Gaussian kernel density estimate) (median −15.8 dB, IQR [−20.0,−8.8] dB; Figure 3d). SF12 accounted for 80.56% of frames, SF7 for 8.93%, and SF8–SF11 jointly for 10.51% (SF8 0.76%, SF9 3.65%, SF10 3.79%, SF11 2.31%; Figure 3b). The three channels at 868.1, 868.3 and 868.5 MHz, which form the mandatory default subset for EU868 devices [6], carried 93.66% of frames (30.80%, 34.82% and 28.05%), while each of the other five channels carried 0.75–1.79% (Figure 3a). Because the gateway does not observe network-server channel plans, this concentration cannot distinguish optional channels that were not configured from configured channels that went unused.

SF7 frames had a mean SNR of −4.95 dB (median −6.20 dB, n=4902) and SF12 frames −15.26 dB (median −17.50 dB, *n* = 44,223), a difference of 10.31 dB (Cohen’s d=1.57, frame-level and descriptive). The naive Welch interval for this difference, [10.16,10.45] dB, was narrower than the day-block bootstrap interval, [9.70,10.89] dB, whose standard error was 4.2 times larger; the difference nonetheless remained well separated from zero. Accounting for dependence changes the estimated uncertainty rather than the point estimate: the SNR difference remains 10.31 dB, while the interval width increases from 0.29 to 1.19 dB. For this mean difference, the squared ratio of the standard errors gives an approximate design effect of 17.6 (4.2^2^); dividing the 49,125 SF7 and SF12 frames by this value gives a rough, heuristic effective sample size of about 2800 frames. This value is specific to this comparison and approximate, because the naive interval is a two-group Welch interval and the bootstrap standard error is itself estimated. Apart from this naive interval and the frame-level intervals shown for comparison in Section 5.5, all reported CIs are based on day-level resampling, so within-day dependence is reflected in them.

Figure 3f shows the SNR distribution of each SF against its demodulation limit (boxes: IQR; whiskers: 1.5 times the IQR; solid line: median): the median SNR of SF12 frames lay 2.5 dB above the SF12 limit, and a substantial upper portion of the SF12 distribution lay above the limits of lower SFs (Section 5.9).

Traffic followed a diurnal cycle (Figure 3e), from a mean of 44.5 frames per hour at 03:00 to 122.1 at 13:00 local time over the 30 complete days. Over the 30 complete days, 61.50% of frames were received between 06:00 and 17:59 local time (day-block 95% CI 58.52–64.56%; per-day range 48.8–70.5%; Wilcoxon W=4.0, $p=1.3\times 10^{-8}$). Daily counts showed no detectable difference between weekdays (mean 1785.0, 22 days) and weekends (mean 1935.3, 8 days; difference 95% CI [−379.3,714.0]; Mann–Whitney U=78.0, p=0.662); with only eight weekend days, this comparison can detect only large differences.

Pairwise Pearson correlations are listed in Table 2. SF correlated negatively with SNR (r=−0.408) and positively with frame size (r=0.419). This association with frame size is a correlational observation; it is largely explained by message-class composition (Section 5.3), and the present data cannot establish a causal link. Frame sizes were concentrated at the 23-byte length of join requests; 23-byte frames accounted for 79.37% of SF12 and 49.43% of SF7 frames; SF7 frames of other sizes were 13–17 bytes. Frames received on radio 0 (867.1–867.9 MHz; 3479 frames) had a median frequency offset of 9953 Hz and included no SF7 or 23-byte frames, against 1232 Hz on radio 1.

### 5.3. Message-Class Composition

Table 3 summarizes the analysis window by message class. Join requests formed 73.44% of frames and 77.73% of observed airtime, data uplinks 22.04% and 22.10%, and frames without a valid LoRaWAN uplink header 4.52% and 0.17%. All 4902 SF7 frames were either join requests (2423) or frames without a valid LoRaWAN uplink header (2479); no SF7 data uplink was observed. Data uplinks used SF8–SF12; SF12 data uplinks (9126 frames) arrived at a median SNR of −19.20 dB, 0.8 dB above the SF12 demodulation limit, whereas SF12 join requests (35,095 frames) arrived at a median SNR of −16.80 dB (upper quartile −9.20 dB).

### 5.4. Supervised Classification

Table 4 lists the search spaces and the selected hyperparameters. Several selected values lie at the boundary of the search space; with 12 draws the search is coarse. LightGBM obtained the highest inner cross-validation PR-AUC (0.9597) and was therefore selected as the primary model. On the chronological test set it reached F1 =0.7917 (95% CI [0.7369,0.8218]), MCC =0.7907, ROC-AUC =0.9946 ([0.9903,0.9997]) and PR-AUC =0.9376 ([0.9102,0.9941]; Table 5). XGBoost (F1 =0.7964) and Random Forest (F1 =0.7891) performed comparably, with overlapping intervals, followed by Extra Trees (0.7578), the SVM (0.5670) and logistic regression (0.3670), whose ROC-AUC (0.9664) nevertheless remained high. Paired day-block differences in F1 between the three ensembles were not resolved (LightGBM minus XGBoost −0.0047, 95% CI [−0.0126,0.0034]; LightGBM minus Random Forest +0.0026, [−0.0075,0.0133]). In PR-AUC, Random Forest was slightly but consistently lower than LightGBM (difference −0.0038, [−0.0065,−0.0013]) and XGBoost (−0.0085, [−0.0145,−0.0020]), whereas the difference between LightGBM and XGBoost was not resolved (−0.0047, [−0.0112,0.0010]). The two gradient-boosting ensembles therefore cannot be ranked against each other on these data. The SNR threshold rule reached F1 =0.3078, and the SNR-only logistic baseline ranked frames moderately well (ROC-AUC =0.8747) but reached only PR-AUC =0.2680 against a no-skill level of 0.0567.

**Figure 4:**
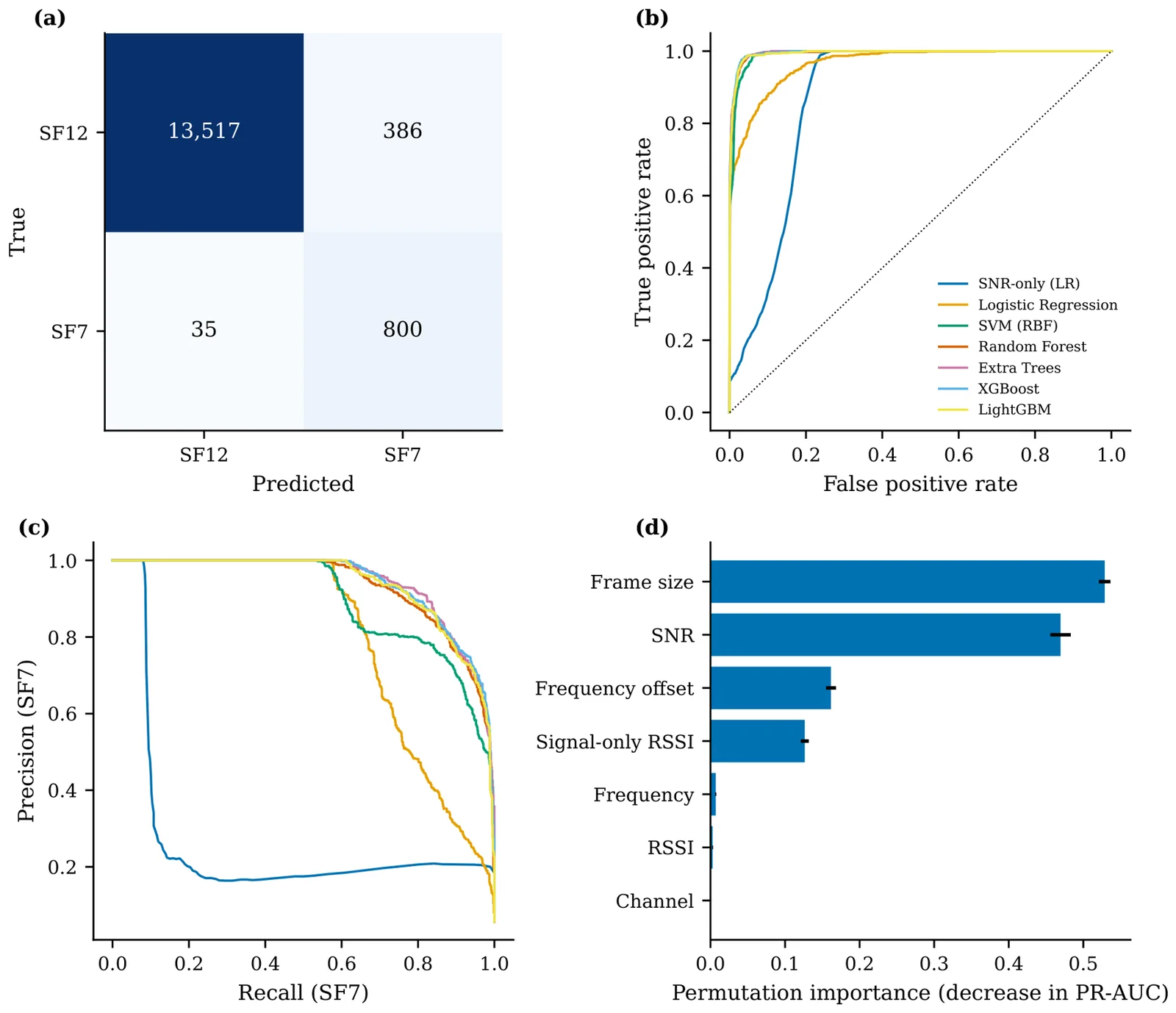
Supervised results on the chronological test set: (**a**) LightGBM confusion matrix; (**b**) receiver operating characteristic (ROC) curves; (**c**) precision–recall curves (colors as in (**b**)); (**d**) permutation importance of the LightGBM features (decrease in PR-AUC; error bars: standard deviation (SD) over ten permutations).

Figure 4 shows the confusion matrix, receiver operating characteristic and precision–recall curves, and permutation importances. Frame size produced the largest decrease in PR-AUC when permuted (0.5287), followed by SNR (0.4695), frequency offset (0.1616) and signal-only RSSI (0.1265); RSSI, frequency and channel contributed less than 0.01 each. SNR thus produced the second-largest decrease in PR-AUC under permutation.

Weekly forward-chaining evaluation (Table 6) gave ROC-AUC of at least 0.9871 for all tree ensembles in every fold. F1 was less stable: LightGBM reached 0.8796 and 0.8631 in weeks 2 and 3, fell to 0.7642 in week 4, when the test week contained only 434 SF7 frames, and logistic regression fell from 0.6648 to 0.3316. The final fold covers only 2.2 days (2993 frames, 129 SF7), so its values are reported for completeness and not interpreted.

### 5.5. Feature Ablation

Table 7 reports the ablation; its F1 intervals are based on 500 day-block resamples, so the all-features interval differs slightly from that in Table 5. Removing the frequency offset reduced LightGBM F1 from 0.7917 to 0.5159 and PR-AUC from 0.9376 to 0.8933; removing frame size reduced F1 to 0.7625 and PR-AUC to 0.8842; removing both reduced F1 to 0.4428 and PR-AUC to 0.7133. With link-quality variables only (SNR, RSSI and signal-only RSSI), LightGBM reached F1 =0.3984 and PR-AUC =0.6806, close to the LightGBM SNR-only result (F1 =0.3247). Frame size alone reached F1 =0.7339, with a wide interval ([0.5046,0.9844]) reflecting day-to-day variation, and frequency offset combined with frame size reached PR-AUC =0.7454, higher than the link-quality variables combined. Because F1 depends on the decision threshold, F1 and PR-AUC occasionally ranked subsets differently (e.g., frame size alone versus frequency offset combined with frame size); PR-AUC, which is threshold-independent, is the more reliable basis for comparison. The same pattern held for logistic regression.

Paired day-block intervals indicated that removing frame size (PR-AUC difference 0.0534, 95% CI [0.0232,0.0985]) and removing the frequency offset (0.0443, [0.0093,0.0758]) each reduced PR-AUC, whereas the difference between frequency offset combined with frame size and the link-quality variables (0.0648, [−0.0763,0.3143]) was not resolved on the 12-day test set. Restricting the analysis to radio-1 frames (F1 =0.7948, PR-AUC =0.9388; without frequency offset 0.5174 and 0.8925) or centering the frequency offset on the training median of each front-end (F1 =0.7972, PR-AUC =0.9383) left these results essentially unchanged, so the contribution of the frequency offset is not explained by the difference between the radio front-ends.

Restricting the analysis by message class changed this picture (Table 8). Within LoRaWAN uplinks, the same model reached PR-AUC =0.6454 (no-skill level 0.0234), link-quality variables alone 0.5010 and frequency offset combined with frame size 0.1129; removing frame size changed PR-AUC by 0.0079 (95% CI [−0.0011,0.0152]) and removing frequency offset by 0.1201 ([−0.0325,0.2153]). Within join requests only, where frame size is constant and the subset without frame size is therefore not reported, the corresponding values were 0.5417, 0.3890 and 0.0859. The intervals are wide because the test sets contained only 328 and 179 SF7 frames; the paired difference between frequency offset combined with frame size and link-quality variables (−0.3881, 95% CI [−0.4674,0.0045] within LoRaWAN uplinks) was therefore not resolved on the 12-day test set.

On the external test period, which contained 322 SF7 LoRaWAN uplinks (no-skill level 0.0083), the same ordering held and was resolved: link-quality variables reached PR-AUC =0.1990 and frequency offset combined with frame size 0.0373 (paired difference −0.1617, [−0.2726,−0.0599]), and removing the frequency offset did not reduce PR-AUC (−0.0215, [−0.0858,0.0482]). The ordering indicates that the contribution of frame size and frequency offset in Table 7, and on the external period in Section 5.7, arises largely from the class of frames without a valid LoRaWAN uplink header, represented by 2479 SF7 frames in the analysis window, which differ from LoRaWAN uplinks in size (mostly 15 or 17 bytes) and channel (868.3 MHz only).

Table 9 compares frame-level and day-block intervals for the classification results. The ratio of the day-block to the frame-level standard error had a median of 2.1 (range 0.9–5.4 across the 16 quantities). For 3 of the 13 paired contrasts, the frame-level interval excluded zero whereas the day-block interval did not: the PR-AUC difference between LightGBM and XGBoost, and the difference between frequency offset combined with frame size and the link-quality variables, both on all frames and within LoRaWAN uplinks in the analysis window. Treating frames as independent would therefore have presented these three differences as resolved, although the day-to-day variation in the data does not support them.

### 5.6. Multi-Class Classification and Temporal Features

The six-class model reached macro-F1 =0.5758 (Random Forest) and 0.5625 (LightGBM), with per-class F1 (Random Forest) of 0.7670 for SF7, 0.3164 for SF8, 0.6385 for SF9, 0.3348 for SF10, 0.4374 for SF11 and 0.9607 for SF12 (Table 10). The test set contained 119 SF8, 706 SF9, 842 SF10 and 375 SF11 frames. The three-class model reached macro-F1 =0.8480 (Random Forest) and 0.8416 (LightGBM). Without the frequency offset, macro-F1 fell to 0.3896 and 0.6293 for the six- and three-class Random Forest models, respectively. In the six-class LightGBM confusion matrix, most SF12 errors were assignments to SF10 (391 frames), SF11 (302) and SF7 (271).

Adding the eleven temporal features to the base features raised LightGBM F1 from 0.7917 to 0.8527 and PR-AUC from 0.9376 to 0.9505; on their own, the temporal features reached F1 =0.1830 and PR-AUC =0.1651. Without the frequency offset, the temporal features raised F1 from 0.5159 to 0.5854. Because frame counts in short windows also rise during transmission bursts of individual devices, this gain may partly reflect device activity patterns rather than temporal signal dynamics. With temporal features included, Isolation Forest flagged a largely different set of frames (Jaccard index 0.251 with the base flags), characterized by higher local traffic density (mean 44.99 frames in the preceding 15 min for flagged versus 25.37 for unflagged frames) and moderately higher signal levels (mean SNR −5.20 dB, RSSI −112.98 dBm).

### 5.7. Temporal Generalization on the External Test Period

The external test period contained 39,710 SF7/SF12 frames, of which only 1051 (2.65%) were SF7, compared with 9.98% of SF7/SF12 frames in the analysis window and 5.67% in the chronological test set. Refitted on the full analysis window, LightGBM reached F1 =0.7161 (95% CI [0.6582,0.7713]), precision 0.6183, recall 0.8506, ROC-AUC =0.9888 ([0.9829,0.9940]) and PR-AUC =0.8603 ([0.7920,0.9163]; no-skill level 0.0265) on this period (Table 11; 95% CIs from 500 day-block resamples). Without the frequency offset, F1 fell to 0.4096 while PR-AUC remained as 0.8528 (paired difference 0.0075, 95% CI [−0.0126,0.0283]); without frequency offset and frame size, F1 fell to 0.2362 and PR-AUC to 0.5099; with SNR only, F1 was 0.1668. Removing frame size reduced PR-AUC by 0.1077 ([0.0311,0.1764]), and frequency offset combined with frame size alone exceeded the link-quality variables alone by 0.2977 ([0.1486,0.4348]); within LoRaWAN uplinks, this advantage reversed (Section 5.5).

**Figure 5:**
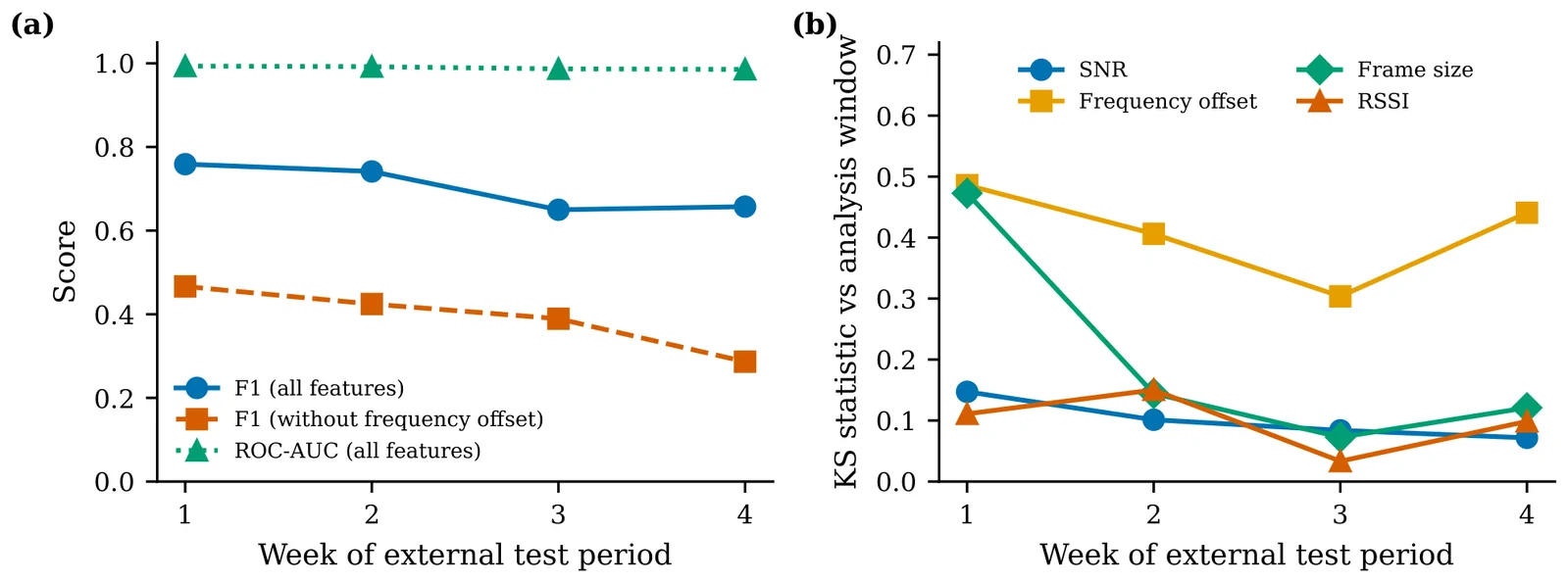
External test period by week: (**a**) LightGBM F1, with and without frequency offset, and ROC-AUC; (**b**) two-sample Kolmogorov–Smirnov (KS) statistic of each feature against the analysis window.

Weekly F1 declined from 0.7589 in the first external week to 0.6574 in the last, while ROC-AUC declined only from 0.9935 to 0.9856 (Figure 5a). Of the four features examined, the frequency offset showed the largest distribution shift relative to the analysis window in every external week (KS statistic 0.3037–0.4860), whereas the shift in SNR decreased from 0.1471 to 0.0717 (Figure 5b). Within SF12 frames, the shift in frequency offset persisted (KS 0.3100–0.5169), whereas that in SNR was small after the first week (0.0219–0.0550, against 0.2174 in week 1). The three-class LightGBM model reached macro-F1 =0.7877 on the external period.

Over the whole external test period (48,176 frames), join requests formed 59.77% of frames and data uplinks 38.72%, against 73.44% and 22.04% in the analysis window. This composition was not stationary: the weekly join-request share of all frames rose from 41.04% in the first external week to 60.49%, 75.84% and 80.22% in the following weeks, while weekly F1 was 0.7589, 0.7415, 0.6499 and 0.6574 and the weekly SF7 share of SF7/SF12 frames was 2.28%, 3.71%, 2.95% and 1.74%. F1 was thus highest in the week whose composition differed most from the analysis window, so join-request share alone does not provide a simple explanation for the decline in F1. With four weekly observations, the inverse association between join-request share and F1 cannot be separated from other changes over time, whereas the frequency-offset shift remained large in every week.

After removal of a linear time trend, the daily median frequency offset of SF12 join requests covaried with outdoor air temperature (Spearman ρ=0.497, 47 days) and with the gateway’s processor temperature (ρ=0.756, 29 days), whereas that of SF12 data uplinks showed weaker, negative associations (ρ=−0.265, 41 days; ρ=−0.372, 24 days). Because consecutive daily values are serially dependent, these coefficients are reported descriptively rather than as significance tests. The median frequency offset of SF12 data uplinks changed from 9440 Hz in the analysis window to 478 Hz in the external period.

### 5.8. Unsupervised Analysis

**Figure 6:**
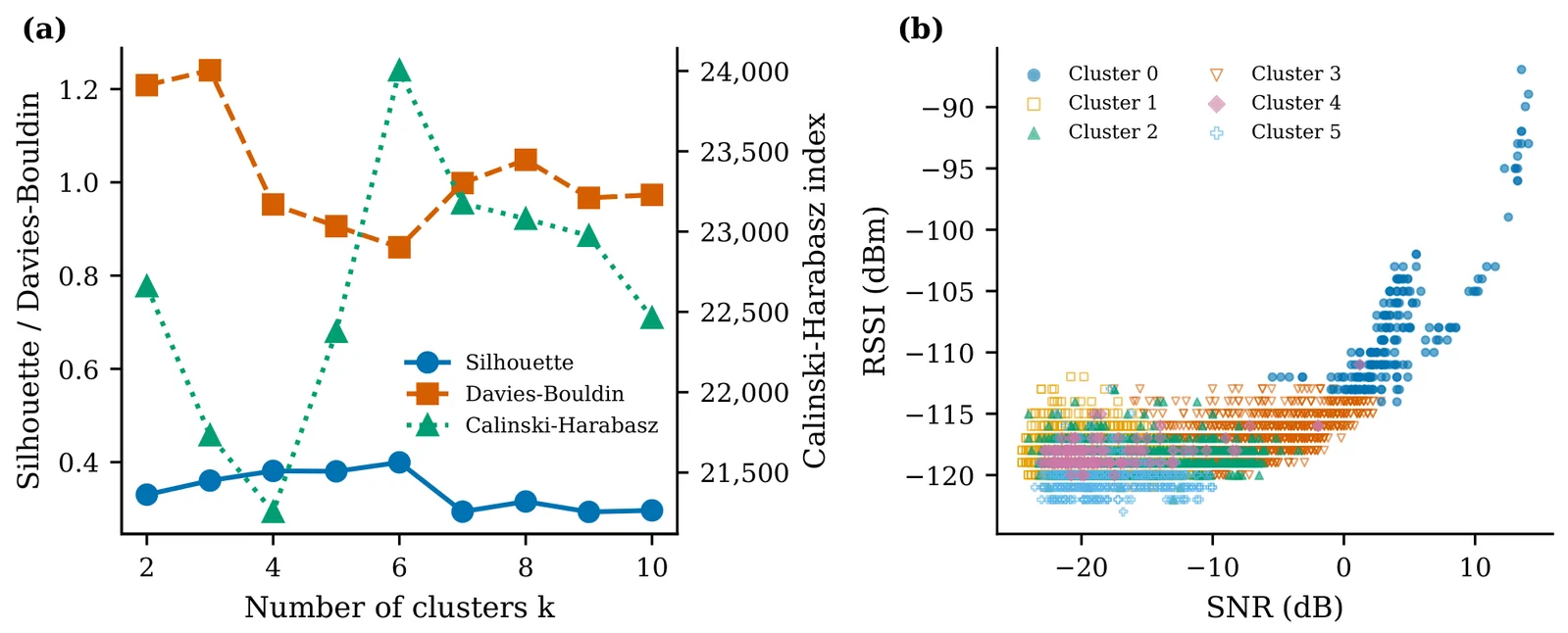
SF-free clustering: (**a**) cluster-validity indices for k=2–10; (**b**) the six clusters on the SNR–RSSI plane. Because the clusters were formed on six features, they partly overlap in this two-dimensional projection.

Over k=2 to 10 (Figure 6a), all three indices reached their optimum at k=6 (silhouette 0.3996, Davies–Bouldin 0.8607, Calinski–Harabasz 24,004.1), an interior point of the search range; k=6 was therefore selected by the pre-specified rule. The partition was stable across random initializations (mean ARI 1.0000, minimum 0.9999). Partitions refitted on individual weeks agreed with the global partition in weeks 1–3 (ARI 0.9392, 0.8888 and 0.9413) but less in weeks 4 and 5 (0.5581 and 0.5422), indicating that the cluster structure itself changed over the window.

Table 12 profiles the six clusters, and Figure 6 shows them on the SNR–RSSI plane. Cluster 1 (48.49% of frames) is a weak-link regime (mean SNR −18.52 dB) containing no SF7 frames. Cluster 3 (27.48%; mean SNR −6.39 dB) contains 84.05% of all SF7 frames, yet SF12 frames form 68.05% of its membership. Clusters 2 and 5 are distinguished mainly by large mean frequency offsets (10,535 and 9782 Hz), and Cluster 5, additionally, by its concentration on the non-mandatory channels (mean 867.58 MHz). Cluster 4 (1.69%) contains frames of 43.3 bytes on average, 92.90% of them SF12, and Cluster 0 (2.73%) contains the strongest signals (mean RSSI −108.68 dBm, mean SNR +3.77 dB).

The share of frames over-provisioned at a 10 dB margin (Section 5.9) differed sharply between clusters: 76.18% in Cluster 0 and 40.78% in Cluster 3, against 3.01% or less in the other four clusters; the two clusters together contained 99.43% of all over-provisioned frames. These two clusters comprised 30.21% of all frames (Table 12), so their share of over-provisioned frames was about 3.3 times their share of frames. This is a descriptive comparison, not an improvement over an SNR-based rule, which identifies over-provisioned frames directly. Because both the clusters and the over-provisioning indicator depend on SNR, this association is partly expected by construction; beyond SNR, the clusters are distinguished mainly by their joint profiles of frequency offset, frame size and channel. Cluster 5 corresponds largely to the radio-0 channels. By message class, Clusters 4 and 5 consisted entirely of data uplinks and Cluster 2 mainly of data uplinks (60.84%); Clusters 1 and 3 consisted predominantly of join requests (87.78% and 87.81%), and Cluster 0 consisted of join requests (76.38%) and frames without a valid LoRaWAN uplink header (23.62%).

For classification, the clustering added no resolved information: adding cluster membership to the selected model left its performance unchanged (PR-AUC 0.9376 on the chronological test set and 0.8603 on the external test period, as without it), and adding the distances to the six centroids changed PR-AUC by +0.0016 (95% CI [−0.0001,0.0031]) and +0.0138 ([−0.0011,0.0304]), respectively. On the chronological test set, the upper 95% confidence limit for the improvement was 0.0031 in PR-AUC; on the external test period, the improvement was not resolved. The clusters are therefore used descriptively.

Isolation Forest flagged 273, 549, 1098 and 2745 frames at contamination values of 0.5%, 1%, 2% and 5%; the number follows from the parameter and is not itself a finding. At 2%, flagged frames had higher RSSI (mean −109.66 versus −118.21 dBm) and SNR (+0.43 versus −14.44 dB), shorter frames (20.70 versus 23.27 bytes) and lower SF (mean 9.92 versus 11.34) than unflagged frames; 785 of the 1098 flagged frames belonged to Cluster 0. The strongest flagged frame reached −72 dBm RSSI and +13.8 dB SNR (20 August 2026, 09:52 UTC). This profile is consistent with transmitters closer to the gateway than the typical population, but transmitter location could not be verified.

### 5.9. Spreading-Factor Over-Provisioning and Airtime

**Figure 7:**
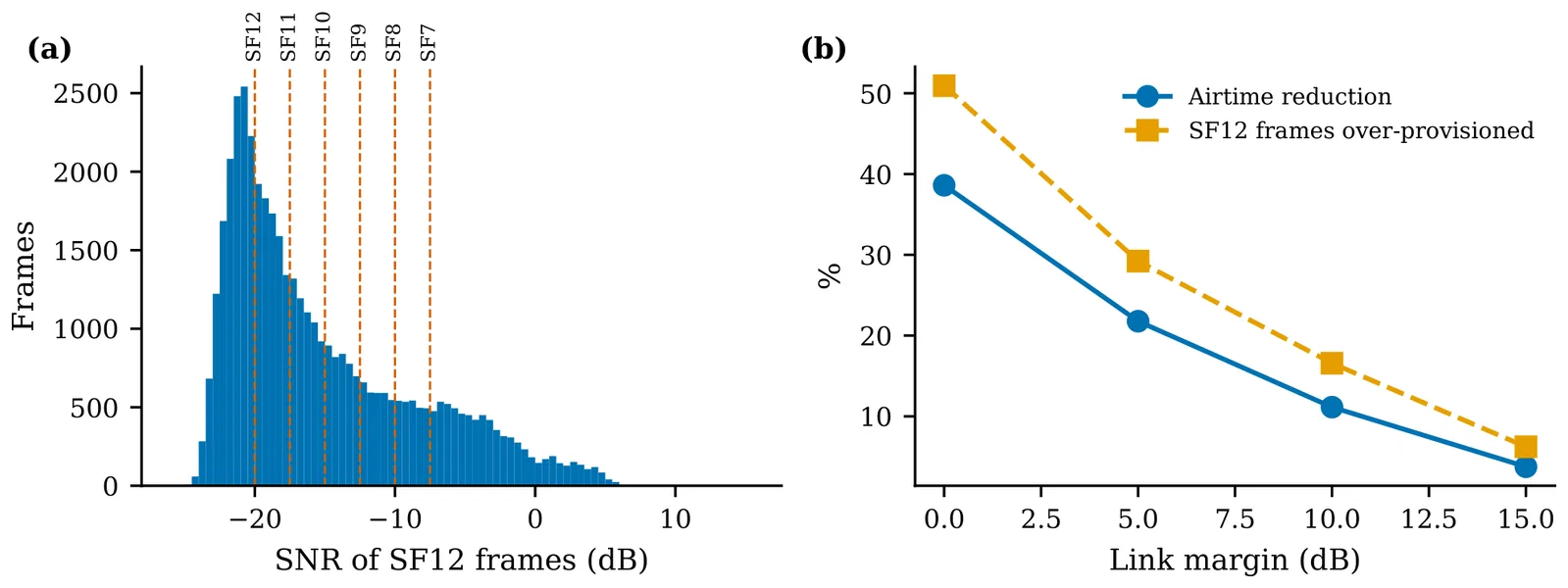
(**a**) SNR of SF12 frames, with dashed lines marking the demodulation limits of SF7–SF12; (**b**) share of over-provisioned SF12 frames and counterfactual airtime reduction versus link margin.

The analysis window contained 68,786.5 s of observed airtime summed over all channels, equivalent to 2.63% of the 725.5 h window duration (aggregate decoded airtime, not channel occupancy; 0.33% per channel on average over the eight channels); SF12 frames accounted for 96.36% of this airtime. Table 13 and Figure 7 report the over-provisioning analysis, computed from Equations (1) and (2). At a 10 dB margin, 16.58% of SF12 frames, and 13.36% of all frames, arrived with SNR sufficient for a lower SF; assigning each such frame the lowest admissible SF would have reduced observed airtime by 11.14% in this counterfactual, corresponding to 7665.0 s (about 2.13 h) of aggregate airtime over the 30.2-day window. The corresponding reductions were 38.61% at 0 dB, 21.78% at 5 dB and 3.76% at 15 dB. Split by message class (Table 14), 20.81% of SF12 join requests but only 0.31% of SF12 data uplinks met the 10 dB criterion, so join requests accounted for 11.08 and data uplinks for 0.06 of the 11.14 percentage points of counterfactual airtime reduction. Even at 5 dB, which is the margin usually used by the Semtech-recommended algorithm, 9.76% of SF12 data uplinks qualified, against 34.28% of SF12 join requests.

## 6. Discussion

### 6.1. What Determines Spreading-Factor Usage in the Observed Traffic

The message-class analysis changes the interpretation of the classifier. On all frames, frame size and frequency offset added discriminative information beyond SNR (Section 5.5), but this contribution arose largely from 2479 SF7 frames without a valid LoRaWAN uplink header, all received at 868.3 MHz, almost all with 15- or 17-byte payloads, and therefore most likely transmitted by LoRa applications that do not use the LoRaWAN uplink format. Within LoRaWAN uplinks, link-quality variables outperformed frame size combined with frequency offset both in the analysis window (PR-AUC 0.5010 against 0.1129) and on the external test period (0.1990 against 0.0373), and frame size contributed little measurable information. Frequency offset combines the crystal-oscillator errors of the transmitter and the gateway, and physical-layer characteristics of LoRa signals have been used to fingerprint individual devices [45]. Its unresolved contribution within LoRaWAN uplinks in the analysis window could reflect information on persistent transmitters; on the external period, removing it did not reduce PR-AUC.

For data uplinks, the observed SF usage is consistent with link-quality-driven assignment: SF12 data uplinks arrived at a median SNR only 0.8 dB above the SF12 limit, and only 0.31% met the 10 dB criterion for a lower SF. This near-limit SNR distribution is the pattern expected from ADR. ADR, however, evaluates SNR at each device’s serving gateway, which the observing gateway generally is not, so agreement at the observing gateway is indicative rather than conclusive. The same distribution would also arise from distant devices statically configured to SF12, because only their frames above the SF12 limit are decoded; the observation therefore does not exclude static configuration.

Join requests behave differently: their data rate follows the join-retry procedure rather than ADR. Repeated join attempts by devices that do not complete the join procedure would explain both the dominance of join requests in frames and airtime and their higher over-provisioning share. Because the observing gateway is not connected to these devices’ networks and device identifiers were excluded, the reasons for repeated join attempts cannot be determined here, and these interpretations remain hypotheses consistent with the data rather than validated mechanisms. The diurnal cycle and the lack of a detectable weekday effect are compatible with autonomous, sensor-driven reporting, but the data do not allow attribution to particular device types.

The same considerations qualify the supervised and temporal results. The binary SF7/SF12 task covers the two extremes of the SF range, so its high discrimination does not imply that adjacent SFs are equally separable; the six-class macro-F1 of 0.5758 indicates that the full six-class task is substantially harder. The SF7 class combined join requests and frames without a valid LoRaWAN uplink header, so the classifier partly learned message class and application rather than link quality, and persistent transmitters may have appeared in both training and test periods. Because no SF7 data uplink was observed, the binary task does not test the SF selection of data uplinks; the evidence for data uplinks rests on the SNR-margin analysis (Section 5.9).

On the external test period, recall was 0.8506 for the model refitted on the full window, compared with 0.9581 for the model trained on the first 70% of the window and evaluated on the chronological test set, alongside a lower SF7 prevalence (2.65% against 5.67% in the chronological test set). ROC-AUC remained high, so the ranking of frames was largely preserved, but the class-conditional feature distributions shifted, most persistently in frequency offset. Two sources of this shift are consistent with the data (Section 5.7). For SF12 join requests, the frequency offset covaried with outdoor and gateway temperature after detrending, as expected from temperature-dependent oscillator error in the transmitters and the gateway. For SF12 data uplinks, no such association remained, and the large change in their median frequency offset is more plausibly explained by a change in the population of transmitting devices. Because the outdoor series is model output rather than an on-site measurement, and processor temperature also depends on computational load, these associations are indicative only.

### 6.2. Application Scenarios

The analysis suggests four practical uses for operators who lack access to the network servers of the networks they observe. All four require only a commercially available gateway with factory calibration and a non-optimized antenna; no software-defined radio, network-server account or payload key is needed. Table 15 lists, for each use, the quantity obtained from the gateway metadata and its value at the present site. First, before a new deployment, a receive-only gateway can quantify the aggregate decoded airtime observed at its location; at the present site, aggregate decoded airtime summed over the eight channels was equivalent to 2.63% of the observation time, with 77.73% of it in join requests. Second, the message type separates two operationally different questions: SF usage of data uplinks can be audited against the link margin (0.31% over-provisioned at 10 dB here), whereas join requests, which consumed most airtime and nearly all counterfactual savings, may indicate devices that repeatedly attempt to join, a condition that operators can investigate once it is detected.

Third, the SF-free clusters provide a descriptive segmentation without any label: the join-dominated Clusters 0 and 3 concentrate over-provisioned frames (76.18% and 40.78% of their frames, together 99.43% of all over-provisioned frames), whereas Clusters 4 and 5 consist of data uplinks. Because the cluster structure changed over the window (weekly ARI 0.5581 and 0.5422 in weeks 4 and 5), the segmentation would require periodic re-estimation if used operationally rather than being applied as a fixed model. Fourth, shifts in message-class composition and in device-related features, such as the frequency-offset KS statistic, can indicate, together with temperature records, that the observed traffic population or operating conditions may have changed and that models or baselines may need to be re-established. On the external test period, both indicators, measured from gateway metadata without payload decoding or device identifiers, changed: the weekly join-request share rose from 41.04% to 80.22%, and the KS statistic of the frequency offset relative to the analysis window remained between 0.3037 and 0.4860 in every week. These values illustrate the four uses at a single site; their benefit for a particular operator has not been evaluated here.

### 6.3. Privacy and Data Minimization

The privacy handling in this study is data minimization rather than a novel privacy-preserving technique: fields that are not needed are not extracted [22]. Minimization limits exposure but does not make the retained metadata anonymous. The ablation indicates that frequency offset and frame size, both retained, carry substantial discriminative information that may correlate with transmitter or application identity, and physical-layer characteristics of LoRa signals are a recognized basis for fingerprinting individual devices [45]. Combined with timestamps, the released metadata could therefore allow frames to be linked to persistent transmitters, although not to device identifiers or owners. The released dataset retains the frequency offset and timestamps at full resolution, so this risk applies to the public data. Coarsening the frequency offset or perturbing timestamps would reduce this risk, at the cost of the analytical utility that the ablation quantifies; the choice between the two is a trade-off that future data releases should state explicitly. The message type, by contrast, is a 3-bit category field and not a direct device identifier, although the timing of join requests could aid the linkage of retries.

### 6.4. Generalization Across Sites and over Time

The external test indicates that threshold-dependent performance, particularly F1, did not remain stable in the subsequent period, even at the same site: for the same refitted model, weekly F1 ranged from 0.7589 in the first external week to 0.6574 in the last. Across sites, the absolute RSSI and SNR distributions depend on building layout, antenna height, distance to transmitters and local interference, and would be expected to shift; the SF mix and the over-provisioning share depend on the local device population and its configuration. The method, comprising gateway-only acquisition, dependence-aware statistics, ablation, external temporal testing and over-provisioning analysis, is not tied to the site, but the specific numbers are. In single-operator networks with ADR enabled and converged for all devices, the join-request share and the over-provisioning of join requests are expected to be lower. In rural deployments with long links, SF12 is likely to be required more often. In dense multi-gateway deployments, the SNR observed at one gateway does not determine the SF chosen on the basis of the best-placed gateway. Multi-site measurement is required to test these expectations (Section 8).

## 7. Limitations

All data were collected by a single gateway at a fixed location in Ankara, Türkiye, over a 30.2-day analysis window and a 24.9-day external test period; the reported distributions characterize this urban radio environment and are not presented as representative of LoRaWAN activity in general. Because SF12 frames remain decodable over a larger distance than SF7 frames, the observed SF composition over-represents high-SF transmitters relative to the local device population.

Because payloads and device identifiers were excluded, the number of distinct transmitters is unknown, device-level independence between training and test periods cannot be verified, and all device-related interpretations (Section 6.1) rest on PHY-layer metadata rather than confirmed identity. The exclusion also precludes identification of network affiliation (e.g., ChirpStack, The Things Network or Helium). Message classes were derived from the unencrypted header pattern and frame length rather than by full LoRaWAN protocol decoding. Per-transmitter signal trajectories cannot be constructed, so the temporal features describe channel-level rather than device-level signal evolution. The SF-free clusters are signal-quality regimes, not device counts. Several selected hyperparameters lay at the boundary of their search spaces (Table 4), and variance across random seeds was not assessed.

The gateway observes only frames it decodes. Frames below its sensitivity, outside its channels or lost to collisions are absent, which truncates the observed SNR distribution at each SF’s demodulation limit and may bias the over-provisioning estimate. That estimate is, moreover, a frame-level counterfactual based on nominal demodulation limits measured at a gateway that is generally not the serving gateway; it ignores the uplink history used by ADR, fading margins, gateway diversity and regulatory duty-cycle constraints, and should be read as an indicator rather than a prediction of achievable savings.

RSSI and SNR reflect the uncalibrated response of the antenna, cable and concentrator as configured, and signal-only RSSI is truncated at a reporting floor of −141 dBm for 3.71% of frames. Coverage-range or path-loss claims therefore cannot be derived from these data.

## 8. Future Work

The measurement will be extended to multiple gateway locations differing in building density, elevation and distance to known infrastructure to test whether the message-class composition, the agreement between SF and link quality for data uplinks, and the dominance of join requests in airtime recur across sites. Cooperation with a network operator would, in addition, provide ground-truth device configuration and ADR state for a subset of transmitters, allowing the hypotheses in Section 6.1 to be tested directly; device-level join histories would also show whether join requests stem from repeated attempts by a limited number of devices. The drift indicators reported here could also be developed into an online monitoring procedure that detects changes in the device population and triggers model re-estimation.

## 9. Conclusions

This study characterized 30.2 days of ambient LoRa traffic observed by a single receive-only gateway in Ankara, Türkiye, using physical-layer metadata and the unencrypted message type only, and assessed temporal generalization on a subsequent external test period of 24.9 days. Join requests formed 73.44% of frames and 77.73% of airtime. SF7 frames arrived at 10.31 dB higher mean SNR than SF12 frames; a LightGBM classifier separated the two with PR-AUC =0.9376, but, within LoRaWAN uplinks, link-quality variables outperformed frame size combined with frequency offset in both the analysis window and the external test period, and the apparent contribution of frame size and frequency offset came largely from frames without a valid LoRaWAN uplink header. At a 10 dB margin, 20.81% of SF12 join requests but only 0.31% of SF12 data uplinks carried an SNR sufficient for a lower SF, so join requests accounted for nearly all of the counterfactual 11.14% airtime reduction.

These results indicate that SF usage of data uplinks at the observing gateway is consistent with link-quality-driven assignment, whereas join requests dominate the airtime of the shared channels. Gateway metadata, together with the message class derived from the unencrypted header, allow these two components to be separated and their airtime to be estimated using commodity hardware, without payload access or network-server cooperation.

These findings matter in three respects. First, they locate the counterfactual airtime reduction by message class: at a 10 dB margin, data uplinks contributed only 0.06 and join requests 11.08 of the 11.14 percentage points. Although this frame-level counterfactual is an indicator rather than a prediction of achievable savings, it suggests that, at this site, airtime reduction would be sought in join-request traffic rather than in the SF assignment of data uplinks. Second, they show that machine learning results on passively observed traffic depend on the message class: on all frames, frame size and frequency offset added substantial discrimination, but their apparent contribution arose largely from frames without a valid LoRaWAN uplink header, and, within LoRaWAN uplinks, link-quality variables reached a higher PR-AUC than these two features (0.5010 versus 0.1129 in the analysis window), an ordering that was resolved on the external test period (0.1990 versus 0.0373). Third, both results were obtained with a commercially available receive-only gateway and without access to the observed networks, so an operator planning a deployment can perform the same type of assessment at its own site, within the decoding coverage of its gateway.

## Figures and Tables

**Table 1:** Comparison of related studies by data source, information required, aim and limitation with respect to the present question.

Study	Data and Scale	Information Used	Aim	Learning Methods	Limitation for the Present Question
Basford et al. [10]	Own city-scale deployment, Southampton; 20 devices, >135,000 messages	Device logs and application-server messages; known transmitters	Long-term reliability and latency	–	No ambient device population
Mašek et al. [11]	Outdoor measurement, Brno; 303 test locations, up to 20 gateways	Operator NS metadata; known transmitter	RSSI/SNR versus distance dataset	–	Controlled transmitter
Aksoy et al. [12]	18-floor building, rooftop gateway	NS data; known end-devices and field tester	Indoor RSSI, SNR and SF comparison	–	Indoor, controlled devices
Grübel et al. [13]	Office building; 390 nodes, 4 gateways, 2 years	Own nodes; signal data and sensor payloads	Network performance; presence sensing	Explanatory and ANN models	Own deployment
Povalač et al. [3]	Passive sniffers in 4 cities, 17.8–42.0 days each	Unencrypted headers incl. DevAddr	Traffic, security, duty-cycle and Class-B beacon analysis	–	Device identifiers; no frame-level SF–SNR-margin analysis
Spadaccino et al. [14]	Public London dataset; 9 gateways, ≈4 months	Gateway-captured headers incl. DevAddr	Behavior analysis	–	Device identifiers; no frame-level SF–SNR-margin analysis
Choi et al. [4]	Passive sniffer at 8 city locations	Headers incl. DevAddr	Deployment landscape, localization, duty-cycle policing	–	Device identifiers
Garlisi et al. [20]	Operator database incl. packets of other operators; 997,183 packets, 2169 devices	Radio and network fields of stored packets	Device profiling, anomaly detection	*k*-means	Operator-side, device-level records
Hosseinzadeh et al. [17]	Handheld mobile-station survey, Glasgow; 3 base stations, 5007 samples	Known transmitters	Propagation (RSSI) modeling for link budget	Linear regression, SVM, KNN, DT, RF, GB, MLP and others	Controlled transmitters
Rao and Sundar [16]	Simulation	–	SF allocation	PSO, XGBoost, DT, RF	Simulated traffic
Esteves et al. [19]	Real gateway data, Lisbon, >500,000 messages; simulation	Frame metadata incl. DevAddr (per-device models)	Intrusion detection	KNN	Device identifiers
Almufareh et al. [18]	Proposed framework; public dataset (3 sensor arrays, 405,184 rows)	Payload sensor values	Anomaly detection, temperature prediction	IF, linear regression, RF	Payload values
This study	Receive-only gateway, Ankara; 30.2 + 24.9 days, 103,069 frames	PHY/MAC metadata; no payload or direct device identifiers retained	SF–link-quality consistency, airtime	Six classifiers, *k*-means, IF	Single site

Abbreviations: device address (DevAddr); medium access control (MAC); physical layer (PHY); spreading factor (SF); network server (NS); received signal strength indicator (RSSI); signal-to-noise ratio (SNR); artificial neural network (ANN); decision tree (DT); gradient boosting (GB); Isolation Forest (IF); *k*-nearest neighbors (KNN); multi-layer perceptron (MLP); particle swarm optimization (PSO); Random Forest (RF); support vector machine (SVM); extreme gradient boosting (XGBoost).

**Table 2:** Pearson correlation matrix of PHY-layer metadata in the analysis window.

	RSSI	SNR	Signal-Only RSSI	Freq. Offset	Frame Size	SF	Frequency
RSSI	1.000	0.588	0.710	−0.146	−0.168	−0.117	0.237
SNR	0.588	1.000	0.982	−0.077	−0.285	−0.408	0.100
Signal-only RSSI	0.710	0.982	1.000	−0.109	−0.275	−0.361	0.151
Freq. offset	−0.146	−0.077	−0.109	1.000	0.066	−0.054	−0.291
Frame size	−0.168	−0.285	−0.275	0.066	1.000	0.419	−0.145
SF	−0.117	−0.408	−0.361	−0.054	0.419	1.000	−0.058
Frequency	0.237	0.100	0.151	−0.291	−0.145	−0.058	1.000

**Table 3:** Composition of the analysis window by message class, derived from the unencrypted three-bit header code and frame length. Abbreviation: Long-Range Wide-Area Network (LoRaWAN).

Message Class	Frames	Frames (%)	Airtime (s)	Airtime (%)	SF7	SF8–SF11	SF12	Median SNR (dB)	Median Size (B)
Join requests	40,312	73.44	53,465	77.73	2423	2794	35,095	−15.5	23
Data uplinks	12,100	22.04	15,202	22.10	0	2974	9126	−17.8	25
No valid LoRaWAN uplink header	2481	4.52	119	0.17	2479	0	2	−4.8	15

**Table 4:** Hyperparameter search spaces and selected values (*p*: number of features). Abbreviations: light gradient boosting machine (LightGBM); radial basis function (RBF).

Model	Search Space	Selected
Logistic Regression	$C\in \{10^{-3},10^{-2},10^{-1},1,10,100\}$ (grid)	C=100
SVM (RBF)	C∈{0.3,1,3,10}; γ∈{scale,0.03,0.1,0.3} (grid, every 4th frame)	C=10, γ=0.03
Random Forest	trees ∈{200,400}; max depth ∈{6,10,16,none}; min leaf ∈{1,5,20}; max features $\in \{\sqrt{p},0.5,1.0\}$ (12 draws)	200 trees, depth none, min leaf 5, max features 0.5
Extra Trees	as Random Forest (12 draws)	200 trees, depth 16, min leaf 5, max features 1.0
XGBoost	trees ∈{200,400,800}; depth ∈{3,5,7}; learning rate ∈{0.03,0.1,0.3}; subsample, column sample ∈{0.7,1}; min child weight ∈{1,5,20} (12 draws)	200 trees, depth 3, rate 0.3, subsample 0.7, column 0.7, min child 1
LightGBM	trees ∈{200,400,800}; leaves ∈{15,31,63}; learning rate ∈{0.03,0.1,0.3}; min child samples ∈{10,50,200}; subsample, column sample ∈{0.7,1}; subsample frequency 1 (12 draws)	200 trees, 31 leaves, rate 0.03, min child 10, subsample 0.7 (frequency 1), column 1.0

**Table 5:** SF7 vs. SF12 classification on the chronological test set (*n* = 14,738; 835 SF7). 95% confidence intervals (CIs) from day-block bootstrap (1000 resamples). No-skill PR-AUC =0.0567. Best values in bold. Abbreviations: F1 score (F1); logistic regression (LR); Matthews correlation coefficient (MCC); area under the precision–recall curve (PR-AUC); area under the receiver operating characteristic curve (ROC-AUC).

Model	Bal. Acc.	Precision	Recall	F1 [95% CI]	MCC	ROC-AUC [95% CI]	PR-AUC
SNR threshold rule	0.7671	0.1967	0.7078	0.3078 [0.2335, 0.3810]	0.3065	–	–
SNR-only (LR)	0.8759	0.2001	0.9892	0.3329 [0.2450, 0.4126]	0.3870	0.8747 [0.8535, 0.9029]	0.2680
RSSI-only (LR)	0.5735	0.0870	0.3976	0.1428 [0.1050, 0.1810]	0.0776	0.6405 [0.5876, 0.7160]	0.1431
SNR + RSSI (LR)	0.8496	0.1876	0.9449	0.3130 [0.2306, 0.3876]	0.3579	0.8852 [0.8709, 0.9061]	0.3040
Logistic Regression	0.8824	0.2268	0.9617	0.3670 [0.2899, 0.4326]	0.4138	0.9664 [0.9406, 0.9969]	0.8001
SVM (RBF)	0.9526	0.3962	**0.9964**	0.5670 [0.4688, 0.6359]	0.5987	0.9918 [0.9830, 0.9988]	0.8957
Random Forest	0.9633	0.6726	0.9545	0.7891 [0.7318, 0.8215]	0.7878	0.9944 [0.9901, 0.9996]	0.9338
Extra Trees	**0.9700**	0.6193	0.9760	0.7578 [0.6864, 0.7996]	0.7621	**0.9955** [0.9927, 0.9994]	**0.9438**
XGBoost	0.9678	**0.6791**	0.9629	**0.7964** [0.7383, 0.8290]	**0.7957**	0.9953 [0.9920, 0.9996]	0.9423
LightGBM (selected)	0.9652	0.6745	0.9581	0.7917 [0.7369, 0.8218]	0.7907	0.9946 [0.9903, 0.9997]	0.9376

**Table 6:** Weekly forward-chaining evaluation (train on weeks 1,…,k, test on week k+1; fixed configurations). Values are F1/ROC-AUC.

Model	Week 2	Week 3	Week 4	Week 5 (2.2 d)
	(*n* = 11,936; 1427 SF7)	(*n* = 14,574; 1612)	(*n* = 9355; 434)	(*n* = 2993; 129)
Logistic Regression	0.6648/0.9632	0.5381/0.9394	0.3316/0.9740	0.3249/1.0000
Random Forest	0.8658/0.9934	0.8356/0.9926	0.7173/0.9938	0.8037/0.9998
Extra Trees	0.8615/0.9907	0.8458/0.9871	0.8299/0.9902	0.8990/0.9998
XGBoost	0.8810/0.9939	0.8709/0.9934	0.7774/0.9950	0.8716/0.9999
LightGBM	0.8796/0.9942	0.8631/0.9942	0.7642/0.9947	0.8543/1.0000

**Table 7:** Feature ablation on the chronological test set. No-skill PR-AUC =0.0567.

	LightGBM			Logistic Regression		
Feature Set	F1 [95% CI]	ROC-AUC	PR-AUC	F1	ROC-AUC	PR-AUC
All features	0.7917 [0.7381, 0.8217]	0.9946	0.9376	0.3670	0.9664	0.8001
Without frequency offset	0.5159 [0.4183, 0.5853]	0.9883	0.8933	0.3608	0.9603	0.7667
Without frame size	0.7625 [0.6966, 0.8028]	0.9923	0.8842	0.3178	0.9089	0.4091
Without both	0.4428 [0.3434, 0.5210]	0.9713	0.7133	0.3122	0.9012	0.3771
Without signal-only RSSI	0.7746 [0.7206, 0.8058]	0.9932	0.9221	0.3661	0.9660	0.7964
SNR + RSSI + signal-only RSSI	0.3984 [0.2970, 0.4802]	0.9634	0.6806	0.3122	0.8937	0.3448
SNR only	0.3247 [0.2363, 0.4028]	0.9138	0.3659	0.3329	0.8747	0.2680
Frequency offset only	0.3108 [0.2232, 0.3802]	0.8759	0.3544	0.0566	0.2303	0.1462
Frame size only	0.7339 [0.5046, 0.9844]	0.8132	0.6063	0.6822	0.8102	0.6061
Frequency offset + frame size	0.4234 [0.3349, 0.4843]	0.9544	0.7454	0.5988	0.9471	0.7251

**Table 8:** SF 7 vs. SF12 classification restricted by message class (LightGBM with the tuned hyperparameters).

Feature Set	F1	PR-AUC [95% CI]	ROC-AUC
*Analysis window*			
LoRaWAN uplinks ($n_{\mathrm{test}}=13,994$; 328 SF7; no-skill 0.0234)			
All features	0.5798	0.6454 [0.0660, 0.7908]	0.9869
Without frequency offset	0.2743	0.5253 [0.0088, 0.6636]	0.9714
Without frame size	0.5728	0.6375 [0.0682, 0.7901]	0.9864
SNR + RSSI + signal-only RSSI	0.2608	0.5010 [0.0080, 0.6438]	0.9685
Frequency offset + frame size	0.1975	0.1129 [0.0195, 0.2276]	0.8880
SNR only	0.2037	0.1805 [0.0062, 0.3406]	0.9388
Join requests only ($n_{\mathrm{test}}=11,256$; 179 SF7; no-skill 0.0159)			
All features	0.4785	0.5417 [0.0335, 0.7432]	0.9831
Without frequency offset	0.1912	0.3931 [0.0061, 0.5722]	0.9638
SNR + RSSI + signal-only RSSI	0.1799	0.3890 [0.0061, 0.5597]	0.9595
Frequency offset + frame size	0.1381	0.0859 [0.0142, 0.1966]	0.8786
SNR only	0.1398	0.1107 [0.0043, 0.2364]	0.9286
*External test period*			
LoRaWAN uplinks ($n_{\mathrm{test}}=38,980$; 322 SF7; no-skill 0.0083)			
All features	0.3242	0.2284 [0.1275, 0.3270]	0.9653
Without frequency offset	0.1481	0.2499 [0.1307, 0.3628]	0.9634
Without frame size	0.3003	0.2071 [0.1140, 0.3037]	0.9617
SNR + RSSI + signal-only RSSI	0.0870	0.1990 [0.0900, 0.3193]	0.9397
Frequency offset + frame size	0.0792	0.0373 [0.0243, 0.0548]	0.8827
SNR only	0.0694	0.0552 [0.0261, 0.1062]	0.9050
Join requests only ($n_{\mathrm{test}}=25,505$; 320 SF7; no-skill 0.0125)			
All features	0.3330	0.2370 [0.1412, 0.3305]	0.9671
Without frequency offset	0.1517	0.2516 [0.1313, 0.3620]	0.9482
SNR + RSSI + signal-only RSSI	0.1470	0.2419 [0.1296, 0.3464]	0.9474
Frequency offset + frame size	0.0800	0.0382 [0.0255, 0.0562]	0.8311
SNR only	0.1043	0.0814 [0.0455, 0.1239]	0.9059

**Table 9:** Frame-level and day-block bootstrap 95% CIs for the classification results (1000 resamples each). SE ratio: day-block standard error (SE) divided by frame-level SE. Δ: paired difference. ^†^ Frame-level CI excludes zero, whereas the day-block CI does not.

Quantity	Estimate	Frame-Level 95% CI	Day-Block 95% CI	SE Ratio
*Chronological test set, all SF7/SF12 frames*				
LightGBM F1	0.7917	[0.7710, 0.8101]	[0.7369, 0.8218]	2.17
LightGBM PR-AUC	0.9376	[0.9263, 0.9487]	[0.9102, 0.9941]	3.47
LightGBM ROC-AUC	0.9946	[0.9933, 0.9958]	[0.9903, 0.9997]	3.65
ΔF1, LightGBM − XGBoost	−0.0047	[−0.0124, 0.0028]	[−0.0126, 0.0034]	1.01
ΔPR-AUC, LightGBM − XGBoost ^†^	−0.0047	[−0.0079, −0.0015]	[−0.0112, 0.0010]	1.95
ΔF1, LightGBM − Random Forest	0.0026	[−0.0053, 0.0105]	[−0.0075, 0.0133]	1.32
ΔPR-AUC, LightGBM − Random Forest	0.0038	[0.0009, 0.0069]	[0.0013, 0.0065]	0.88
ΔF1, XGBoost − Random Forest	0.0073	[−0.0001, 0.0151]	[−0.0006, 0.0148]	0.95
ΔPR-AUC, XGBoost − Random Forest	0.0085	[0.0047, 0.0127]	[0.0020, 0.0145]	1.59
ΔPR-AUC, all features − without frequency offset	0.0443	[0.0331, 0.0552]	[0.0093, 0.0758]	3.04
ΔPR-AUC, all features − without frame size	0.0534	[0.0357, 0.0715]	[0.0232, 0.0985]	2.02
ΔPR-AUC, frequency offset + frame size − link quality ^†^	0.0648	[0.0278, 0.0994]	[−0.0763, 0.3143]	5.38
*Chronological test set, LoRaWAN uplinks*				
ΔPR-AUC, frequency offset + frame size − link quality ^†^	−0.3881	[−0.4455, −0.3335]	[−0.4674, 0.0045]	3.22
*External test period, all SF7/SF12 frames*				
ΔPR-AUC, all features − without frequency offset	0.0075	[−0.0022, 0.0173]	[−0.0126, 0.0283]	2.08
ΔPR-AUC, all features − without frame size	0.1077	[0.0923, 0.1230]	[0.0311, 0.1764]	4.80
ΔPR-AUC, frequency offset + frame size − link quality	0.2977	[0.2607, 0.3318]	[0.1486, 0.4348]	3.99

**Table 10:** Multi-class SF classification on the chronological test set (macro-F1 and per-class F1).

Scheme	Model/Features	Macro-F1	Per-Class F1					
			SF7	SF8	SF9	SF10	SF11	SF12
6-class	Random Forest/all	0.5758	0.7670	0.3164	0.6385	0.3348	0.4374	0.9607
6-class	LightGBM/all	0.5625	0.7714	0.3120	0.6347	0.3112	0.3933	0.9524
6-class	Random Forest/without freq. offset	0.3896	0.5075	0.1607	0.5811	0.1928	0.1076	0.7879
6-class	LightGBM/without freq. offset	0.3754	0.5219	0.1410	0.5736	0.1866	0.1090	0.7201
			SF7	SF8–SF11				SF12
3-class	Random Forest/all	0.8480	0.7534	0.8265				0.9641
3-class	LightGBM/all	0.8416	0.7562	0.8087				0.9598
3-class	Random Forest/without freq. offset	0.6293	0.4817	0.5557				0.8504
3-class	LightGBM/without freq. offset	0.6135	0.4840	0.5262				0.8303

**Table 11:** Performance on the external test period. No-skill PR-AUC =0.0265.

Model	Features	F1 [95% CI]	MCC	ROC-AUC [95% CI]	PR-AUC
LightGBM	All	0.7161 [0.6582, 0.7713]	0.7167	0.9888 [0.9829, 0.9940]	0.8603
LightGBM	Without frequency offset	0.4096 [0.3598, 0.4608]	0.4784	0.9888 [0.9837, 0.9933]	0.8528
LightGBM	Without freq. offset and frame size	0.2362 [0.1602, 0.3396]	0.3239	0.9578 [0.9434, 0.9711]	0.5099
LightGBM	SNR only	0.1668 [0.1125, 0.2367]	0.2546	0.8972 [0.8608, 0.9242]	0.1952
Logistic Regression	All	0.3154 [0.2725, 0.3633]	0.3895	0.9708 [0.9546, 0.9840]	0.8003
Logistic Regression	SNR only	0.1855 [0.1328, 0.2492]	0.2703	0.8882 [0.8609, 0.9097]	0.1890

**Table 12:** Profiles of the six SF-free clusters. SF shares are post hoc. Over-prov.: share of frames over-provisioned at a 10 dB margin.

Cluster	Share (%)	RSSI (dBm)	SNR (dB)	Freq. Offset (Hz)	Freq. (MHz)	Size (B)	SF7 (%)	SF12 (%)	Over-Prov. (%)
0	2.73	−108.68	3.77	2501	868.30	21.1	23.75	76.25	76.18
1	48.49	−118.63	−18.52	1214	868.29	22.8	0.00	88.85	0.00
2	13.33	−118.56	−15.50	10,535	868.30	23.9	5.82	67.92	0.19
3	27.48	−117.17	−6.39	1587	868.30	22.1	27.31	68.05	40.78
4	1.69	−118.39	−18.69	653	868.24	43.3	0.00	92.90	3.01
5	6.27	−120.21	−18.03	9782	867.58	25.5	0.00	96.77	0.00

**Table 13:** Frame-level SF over-provisioning and counterfactual airtime at four link margins (reductions only; observed airtime 68,786.5 s).

Margin (dB)	SF12 Frames Over-Prov. (%)	All Frames Over-Prov. (%)	Counterfactual Airtime (s)	Airtime Reduction (%)
0	50.94	43.86	42,228.4	38.61
5	29.22	24.31	53,801.7	21.78
10	16.58	13.36	61,121.5	11.14
15	6.23	5.02	66,199.1	3.76

**Table 14:** Over-provisioning by message class (reductions only). The last column gives each class’s contribution to the reduction in total observed airtime in percentage points.

Message Class	Margin (dB)	SF12 Frames	SF12 Over-Prov. (%)	Class Airtime Reduction (%)	Contribution to Total (pp)
Join requests	0	35,095	54.83	43.41	33.74
Join requests	5	35,095	34.28	26.63	20.70
Join requests	10	35,095	20.81	14.26	11.08
Join requests	15	35,095	7.82	4.82	3.75
Data uplinks	0	9126	36.01	22.03	4.87
Data uplinks	5	9126	9.76	4.92	1.09
Data uplinks	10	9126	0.31	0.28	0.06
Data uplinks	15	9126	0.09	0.07	0.01

**Table 15:** Application scenarios, the quantity obtained from gateway metadata, and its value at the present site.

Use	Quantity from Gateway Metadata	Value at the Present Site	Section
Pre-deployment airtime survey	Aggregate decoded airtime and its share by message class	2.63% of the observation time (0.33% per channel); 77.73% in join requests	Section 5.3 and Section 5.9
SF usage audit by message class	Share of SF12 frames with SNR sufficient for a lower SF at a 10 dB margin; counterfactual airtime reduction	Data uplinks 0.31%, join requests 20.81%; 11.08 of 11.14 percentage points of reduction from join requests	Section 5.9
Label-free traffic segmentation	Over-provisioned share by cluster; weekly stability of the clusters	Clusters 0 and 3: 76.18% and 40.78% (99.43% of all over-provisioned frames); other clusters ≤3.01%; weekly adjusted Rand index 0.5581 and 0.5422 in weeks 4 and 5	Section 5.8
Monitoring of population change	Weekly join-request share; Kolmogorov–Smirnov statistic of the frequency offset against the analysis window	Join-request share 41.04% to 80.22% over the external weeks; Kolmogorov–Smirnov statistic 0.3037–0.4860 in every week	Section 5.7

## Data Availability

The processed metadata analyzed in this study, covering the analysis window and the external test period, are publicly available on Kaggle as version 2 of the dataset under a Creative Commons Attribution 4.0 International (CC BY 4.0) license [21], together with the analysis script. Each record carries its message class; payload content and direct device identifiers are not included. The outdoor air temperature series used in Section 5.7 is publicly available from the Open-Meteo service [34]; the gateway processor temperature log is available from the corresponding author on reasonable request.

## Abbreviations

The following abbreviations are used in this manuscript:

ADR	Adaptive Data Rate
ARI	Adjusted Rand Index
CI	Confidence Interval
CRC	Cyclic Redundancy Check
DevAddr	Device Address
EU868	European 863–870 MHz Band
IQR	Interquartile Range
KS	Kolmogorov–Smirnov
LightGBM	Light Gradient Boosting Machine
LoRa	Long-Range
LoRaWAN	Long-Range Wide-Area Network
MAC	Medium Access Control
MCC	Matthews Correlation Coefficient
MType	Message Type
PHY	Physical Layer
PR-AUC	Area Under the Precision–Recall Curve
ROC-AUC	Area Under the Receiver Operating Characteristic Curve
RSSI	Received Signal Strength Indicator
SF	Spreading Factor
SNR	Signal-to-Noise Ratio
SVM	Support Vector Machine
UTC	Coordinated Universal Time
XGBoost	Extreme Gradient Boosting

## Appendix A. Daily Frame Counts

**Table A1:** Daily decoded-frame counts in the analysis window (local calendar dates, Coordinated Universal Time (UTC)+3); the first and last dates are partial.

Date (Local)	Frames	SF7	SF8–SF11	SF12
9 August 2026	82	10	2	70
10 August 2026	1213	196	23	994
11 August 2026	1283	187	20	1076
12 August 2026	1623	276	17	1330
13 August 2026	1551	146	44	1361
14 August 2026	1595	158	52	1385
15 August 2026	1931	218	35	1678
16 August 2026	1331	122	28	1181
17 August 2026	1925	492	29	1404
18 August 2026	1576	143	30	1403
19 August 2026	1673	122	40	1511
20 August 2026	1858	232	45	1581
21 August 2026	1791	163	35	1593
22 August 2026	1683	180	49	1454
23 August 2026	1718	95	50	1573
24 August 2026	2106	154	92	1860
25 August 2026	2156	325	42	1789
26 August 2026	2206	290	166	1750
27 August 2026	2841	169	671	2001
28 August 2026	3998	220	1397	2381
29 August 2026	3526	292	1502	1732
30 August 2026	2570	161	949	1460
31 August 2026	1459	156	71	1232
1 September 2026	1382	60	26	1296
2 September 2026	1437	43	24	1370
3 September 2026	1271	32	11	1228
4 September 2026	1251	18	25	1208
5 September 2026	1344	79	31	1234
6 September 2026	1379	35	15	1329
7 September 2026	1411	64	23	1324
8 September 2026	1664	64	212	1388
9 September 2026	59	0	12	47
Total	54,893	4902	5768	44,223
